# Siglec‐G Suppresses CD8^+^ T Cells Responses through Metabolic Rewiring and Can be Targeted to Enhance Tumor Immunotherapy

**DOI:** 10.1002/advs.202403438

**Published:** 2024-10-07

**Authors:** Shenhui Yin, Chunzhen Li, Xin Shen, Guanyu Yu, Likun Cui, Yunyang Wu, Yixian He, Shu Yu, Jie Chen, Shaoteng Lu, Guifang Qiu, Mengqi Song, Cheng Qian, Zui Zou, Yizhi Yu, Sheng Xu

**Affiliations:** ^1^ National Key Laboratory of Immunity & Inflammation Naval Medical University/Second Military Medical University Shanghai 200433 China; ^2^ Department of Colorectal Surgery Changhai Hospital Naval Medical University Shanghai 200433 China; ^3^ School of Anesthesiology Naval Medical University Shanghai 200433 China; ^4^ Faculty of Anesthesiology Changhai Hospital Naval Medical University Shanghai 200433 China

**Keywords:** CD8^+^ T cells, metabolic rewiring, Siglec‐G, tumor immunotherapy

## Abstract

CD8^+^ T cells play a critical role in cancer immune‐surveillance and pathogen elimination. However, their effector function can be severely impaired by inhibitory receptors such as programmed death‐1 (PD‐1) and T cell immunoglobulin domain and mucin domain‐3 (Tim‐3). Here Siglec‐G is identified as a coinhibitory receptor that limits CD8^+^ T cell function. Siglec‐G is highly expressed on tumor‐infiltrating T cells and is enriched in the exhausted T cell subset. Ablation of Siglec‐G enhances the efficacy of adoptively transferred T cells and chimeric antigen receptor (CAR) T cells in suppressing solid tumors growth. Mechanistically, sialoglycan ligands, such as CD24 on tumor cells, activate the Siglec‐G‐SHP2 axis in CD8^+^ T cells, impairing metabolic reprogramming from oxidative phosphorylation to glycolysis, which dampens cytotoxic T lymphocyte (CTL) activation, expansion, and cytotoxicity. These findings discover a critical role for Siglec‐G in inhibiting CD8^+^ T cell responses, suggesting its potential therapeutic effect in adoptive T cell therapy and tumor immunotherapy.

## Graphical abstract

Siglec‐G is highly expressed on tumor‐infiltrating T cells and activated T cells. The Sialic acids on tumor cells activate the Siglec‐G‐SHP2 axis in CD8^+^ T cells, impairing the metabolic reprogramming from oxidative phosphorylation to glycolysis and consequently dampening CTL activation, expansion, and cytotoxicity. Ablation of Siglec‐G enhances the efficacy of CAR T cells in repressing the growth of solid tumors.

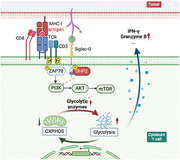

## 1 Introduction

CD8^+^ T cells play important roles in immunosurveillance of malignancies and pathogen infections. However, due to the immunosuppressive nature of tumor microenvironments, tumor‐infiltrating T cells become “exhausted” or otherwise suppressed, severely impairing their proliferative capacity and effector function.^[^ ^1^, ^2^ ^]^ Recent efforts to reactivate immune responses by blocking T cell coinhibitory receptors such as programmed death 1 (PD‐1) or cytotoxic T‐lymphocyte antigen 4 (CTLA‐4) have shown clinical promise.^[^ ^3^, ^4^ ^]^ Meanwhile, pathogens such as bacteria, viruses, fungi, or protozoa can cause acute and chronic infections in their hosts. Effective methods to enhance CD8^+^ T cell‐mediated cytotoxicity against infections have also attracted considerable attention.^[^ ^5^, ^6^ ^]^ Despite the clinical success of immune checkpoint inhibitor therapies, many patients fail to respond or develop resistance over time after an initially encouraging response.^[^ ^7^ ^]^ Recently, the combined blockade of PD‐1 and CTLA‐4 or lymphocyte activation gene 3 (LAG‐3) has demonstrated enhanced antitumor activity greater than the blockade of either receptor alone.^[^ ^8^, ^9^ ^]^ Therefore, there is substantial interests in identifying additional inhibitory receptors expressed on CD8^+^ T cells that contribute to cancer immunesurveillance and pathogen elimination.

Hypersialylation is a hallmark of cancer, with the outer surface of cancer cells frequently coated in a dense layer of sialic acids.^[^ ^10^ ^]^ By recognizing specific sialic acid‐containing glycans (sialoglycans), the Siglec (sialic acid‐binding immunoglobulin‐like lectins) receptors elicit an inhibitory signaling cascade on innate immune cells, including NK cells, macrophages, and neutrophils. This process is mediated by Src homology 2 domain‐containing protein tyrosine phosphatases SHP‐1 and/or SHP‐2.^[^ ^11^, ^12^, ^13^ ^]^ Siglec‐G (the homolog of human Siglec‐10), a member of the Siglec family, is an inhibitory receptor primarily expressed on B cells, where it limits the expansion and calcium signaling of the B1 cell population.^[^ ^14^ ^]^ By targeting Siglec‐G, neutrophils disrupt B‐1a cell homeostasis, exacerbating sepsis.^[^ ^15^ ^]^ Siglec‐G is also expressed on macrophages, inhibiting its innate immune responses.^[^ ^16^ ^]^ Blocking the interaction between Siglec‐G on macrophages and CD24 on tumor cells robustly enhances the macrophage phagocytosis of tumor cells.^[^ ^17^ ^]^ Additionally, Siglec‐G is expressed on dendritic cells and inhibits cross‐presentation, thereby suppressing adoptive immunity.^[^ ^18^ ^]^ Siglec‐10 expression is also associated with NK cell dysfunction and decreases survival in patients with hepatocellular carcinoma.^[^ ^19^ ^]^ However, the role of Siglec‐G in T cells, especially CD8^+^ T cells, remains to be fully elucidated.

Stimulation of the T cell antigen receptor (TCR) and subsequent ligation of costimulatory receptors induce activation and proliferation of naïve CD8^+^ T cells. However, recent advances have highlighted the equally critical reprogramming of cellular metabolism.^[^ ^20^, ^21^ ^]^ Naïve CD8^+^ T cells primarily rely on oxidative phosphorylation (OXPHOS), whereas activated CD8^+^ T cells would switch to glycolytic metabolism to support cell growth and population expansion.^[^ ^22^, ^23^ ^]^ This metabolic reprogramming during the transition from quiescence to activation satisfies the heightened metabolic demands and sustains the functions of CD8^+^ T cells.^[^ ^24^, ^25^, ^26^ ^]^ Despite the importance of glycolytic metabolism in CD8^+^ T cell activation and effector function, the regulatory role of Siglecs, particularly Siglec‐G, in CD8^+^ T cell glycolysis remains unclear.

In this study, we identified Siglec‐G as an inhibitory receptor co‐expressed with checkpoint receptors on CD8^+^ T cells. Activation of SHP‐2 through the sialic acid‐Siglec‐G axis negatively regulated the PI3K‐AKT signaling pathway, thereby dampening the metabolic shift from OXPHOS to glycolysis in activated CD8^+^ T cells. This resulted in reduced CD8^+^ T cell efficacy in both tumor and infection contexts. Targeting Siglec‐G can enhance the tumor‐repressing efficacy of adoptively transferred T cells and chimeric antigen receptor (CAR) T cells.

## 2 Results

### 2.1 Characterization of Siglec‐G Expression on CD8^+^ T Cells

**Figure 1:**
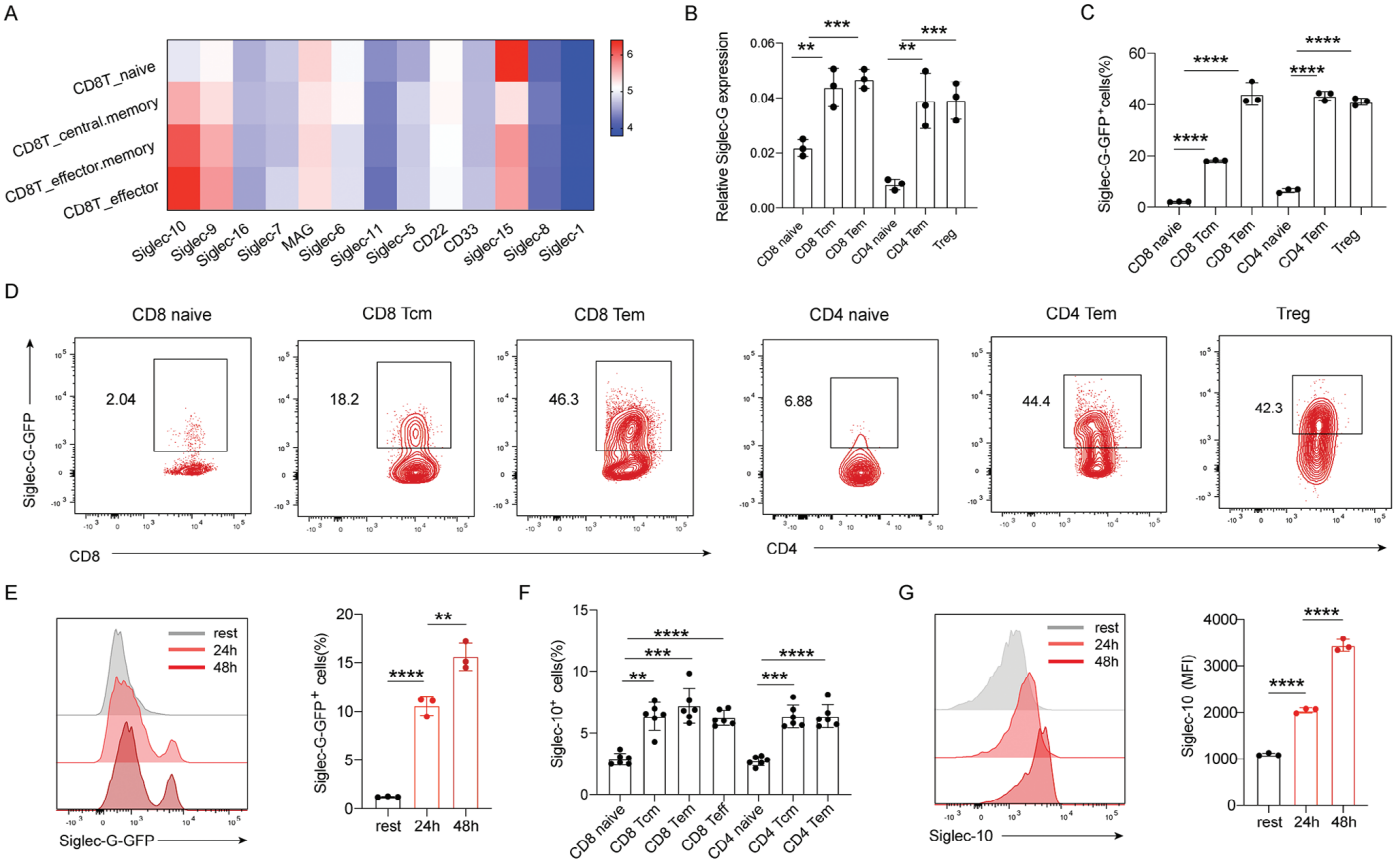
Characteristic expression of Siglec‐G in T cells. A) Heatmap showing Siglecs family genes expression in four CD8^+^ T cells clusters from peripheral blood of healthy volunteers from GEO DataSet (GSE93776). B) Q‐PCR analysis of Siglec‐G expression in murine splenic immune cells sorted from WT mice (*n *= 3). C,D) Siglec‐G‐GFP expression on different murine splenic immune cells (*n* = 3). E) Murine naïve CD8+ T cells were activated with BMDCs supplemented with 100 µg ml^−1^ OVA and Siglec‐G expression was detected over time. F) Siglec‐10 expression on different human T cells from healthy donors (*n *= 6). G) Human CD8^+^ T cells were activated with Human CD3/CD28 stimulation beads and Siglec‐10 expression was detected over time. Representative data are shown from three independent experiments. The error bar represents mean ± SD. Statistical significance was determined by one‐way ANOVA with multiple comparisons (B,C,E–G). **p < 0.01, ***p < 0.001, and ****p < 0.0001.

To investigate the role of Siglecs in T cells, we examined the expression of Siglecs family genes in CD8^+^ T cells isolated from the peripheral blood of healthy volunteers (GSE93776) (**Figure** **1**A). We found that Siglec‐10 and Siglec‐9 were significantly expressed in memory cells (TM), but not in naïve T cells. Further analysis using the Immunological Genome Project database showed that only Siglec‐G, the homologue of human Siglec‐10, exhibited a similar expression profile in mouse (Figure S1A, Supporting Information). Consequently, we focused our study on mouse Siglec‐G.

To verify the expression of Siglec‐G, we isolated different types of immune cells from murine spleens by fluorescence‐activated cell sorting (Figure S1B, Supporting Information). Consistent with previous studies,^[^ ^14^ ^]^ Siglec‐G was highly expressed on B cells (Figure 1B). Higher expression levels of Siglec‐G were observed on memory T cells compared with naïve T cells, which exhibited the lowest levels of Siglec‐G. Flow cytometry analysis using *Siglecg^Gfp/+^* reporter mice, in which GFP serves as a marker for Siglec‐G expression (Figure S2A, Supporting Information), confirmed the elevated Siglec‐G expression on memory‐phenotype T cells, particularly on effector memory CD8^+^ T cells (Figure 1C,D). Additionally, we assessed Siglec‐G expression on T cells using in vivo ovalbumin (OVA)‐expressing *L. monocytogenes* (LM‐OVA) infection models, which also confirmed higher levels of Siglec‐G on effector and memory antigen‐specific T cells (Figure S2B, Supporting Information). Furthermore, Siglec‐G was upregulated on CD8^+^ T cells upon in vitro activation (Figure 1E) as well as on effector CD4^+^ T cells (Figure S2C, Supporting Information).

We then characterized Siglec‐10 expression on human T cells from healthy donors by flow cytometry (Figure S1C, Supporting Information). In line with murine data, naïve T cells exhibited the lowest levels of Siglec‐10, while memory T cells displayed high levels (Figure 1F). In vitro activation also increased Siglec‐10 expression on human CD8^+^ T cells (Figure 1G). Therefore, Siglec‐G can be induced on CD8^+^ T cells and may be sustained till memory T cells, indicating its potential role in T cell responses.

### 2.2 Siglec‐G^+^ CD8^+^ T Cells Exhibit a Suppressed Function

**Figure 2:**
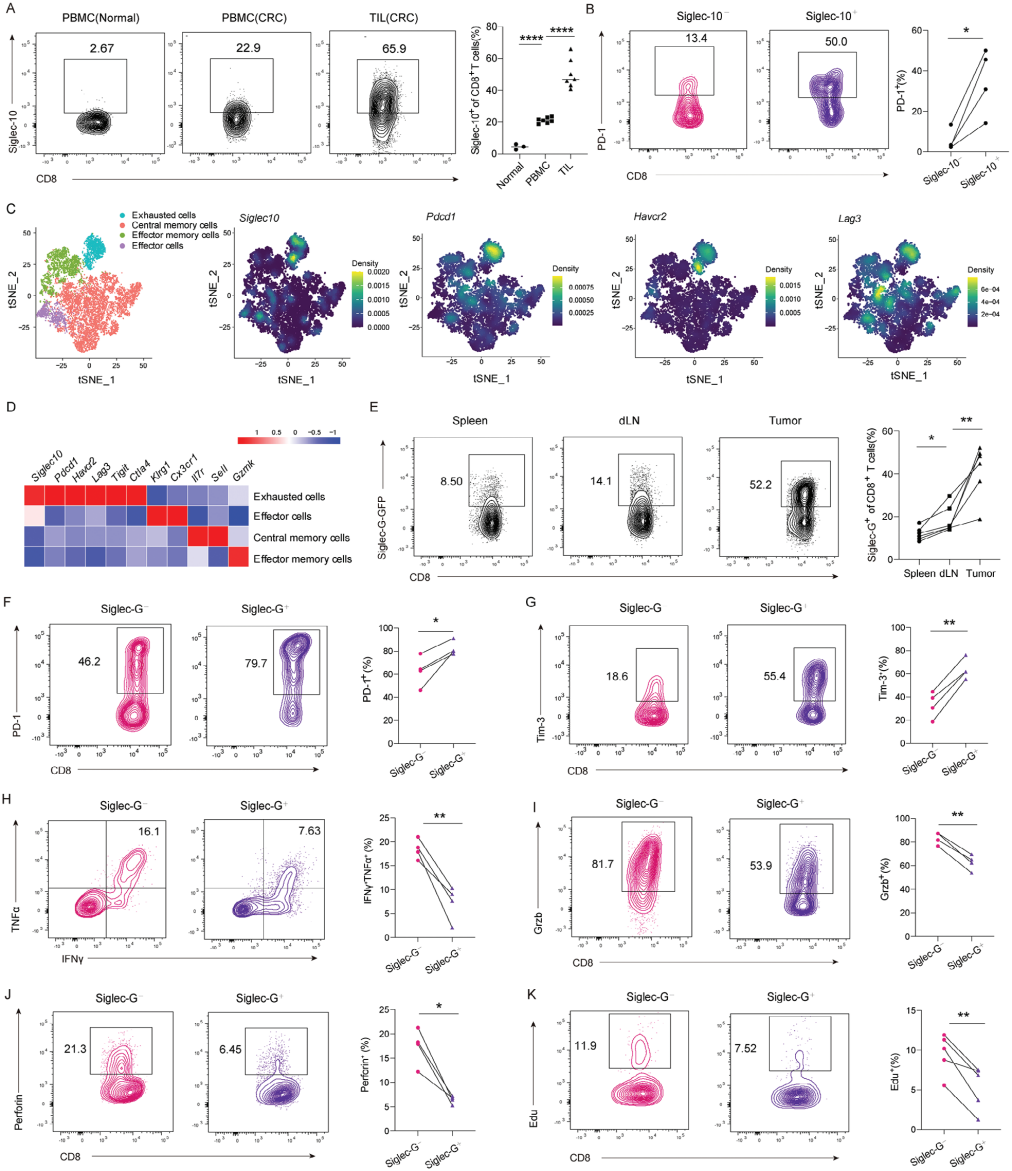
Siglec‐G(Siglec‐10) is coordinately expressed with exhausting markers in tumor‐infiltrating lymphocytes. A) Siglec‐10 expression on CD8^+^ T lymphocytes from healthy donor peripheral blood, tumor‐matched peripheral blood, and human CRC tumors (*n* = 3–7). B) PD‐1 expression on Siglec‐10^−^ and Siglec‐10^+^ tumor‐infiltrating CD8^+^ cells (*n* = 4). C) Plots showing four clusters of intratumor CD8^+^ T cells and the rank‐normalized expression of *Siglec‐10* and *Pdcd1, Havcr2, Lag3* on tumor‐infiltrating single CD8^+^ T cells from GEO DataSet (GSE231559). D) Heatmap of mean expression of T cell function‐associated genes on each cell cluster. E) Siglec‐G expression on splenocytes, dLNs, and tumor‐infiltrating CD8^+^ T cells in *Siglecg^Gfp+^* reporter mice inoculated with MC38 (*n* = 6). F,G) PD‐1(F) and Tim‐3(G) expression in the Siglec‐G^−^ and Siglec‐G^+^ tumor‐infiltrating CD8^+^ cells (*n* = 4,5), H–K) Intracellular IFN‐γ and TNF‐α co‐expression (H), Granzyme B (I), Perforin (J), and fast proliferative proportion (K) in the Siglec‐G^−^ and Siglec‐G^+^ tumor‐infiltrating CD8^+^ cells (*n* = 4,5). Representative data are shown from three independent experiments. The error bar represents mean ± SD. Statistical significance was determined by one‐way ANOVA with multiple comparisons (A and E) and paired Student's *t*‐test (B and F–K). *p < 0.05, **p < 0.01, and ****p < 0.0001.

We then examined the expression of Siglec‐10 on tumor‐infiltrating lymphocytes in human colorectal cancer (CRC). A significant percentage of tumor‐infiltrating CD8^+^ and CD4^+^ T cells expressed Siglec‐10 (**Figure** **2**A; Figure S3A, Supporting Information). Notably, peripheral CD8^+^ and CD4^+^ T cells from CRC donors also showed higher levels of Siglec‐10 compared to those from healthy donors (Figure 2A; Figure S3A, Supporting Information). Furthermore, the expression of PD‐1 was elevated on Siglec‐10^+^ CD8^+^ cells compared to Siglec‐10^−^ counterpart (Figure 2B), indicating a reduced functional capacity of the Siglec‐10^+^ T cells. Consistent with these findings, single‐cell RNA sequencing data of immune cells from early CRC patients revealed that Siglec‐10 was primarily expressed on the exhausted cluster of CD8^+^ tumor infiltrating lymphocytes (TILs), characterized by inhibitory gene expression (*Pdcd1, Havcr2, Tigit*, and *Lag3*) (Figure 2C,D; Figure S3B,C, Supporting Information). However, a subset of Siglec10^+^PD‐1^−^ cells existed independently within the exhausted CD8^+^ T cells (Figure 2C; Figure S3D, Supporting Information), suggesting that the expression patterns of Siglec‐10 and PD‐1 did not completely overlap. Tumor Immune Estimation Resource (TIMER) analysis also indicated a positive association between Siglec‐10 expression and both CD8 and PD‐1 in human cancers (Figure S3E,F, Supporting Information). Taken together, these findings indicate that Siglec‐10 was enriched in tumor‐infiltrating T cells and may represent a suppressed subpopulation or state in tumor immunity.

We further characterized the expression of Siglec‐G in murine MC38 tumor models using *Siglecg^Gfp/+^* reporter mice. Siglec‐G was expressed by ≈50% of CD8^+^ TILs and 30% of CD4^+^ TILs, which was higher than its expression in spleen and dLNs (Figure 2E; Figure S4A, Supporting Information). Similar results were obtained in murine B16 tumor models (Figure S4B, Supporting Information). Consistent with the Siglec‐10 expression in human CRC, CD8^+^ TILs expressing Siglec‐G also exhibited higher levels of PD‐1 and Tim‐3 (encoded by the *Havcr2* gene) (Figure 2F,G). Notably, not all Siglec‐G^+^ CD8^+^ T cells concurrently expressed PD‐1 or Tim‐3 (Figure S4C, Supporting Information), indicating that Siglec‐G is a novel receptor on exhausted T cells, which is not fully overlapped with PD‐1. Furthermore, Tex cells are heterogeneous and include progenitor and terminal subsets with unique characteristics and responses to checkpoint blockade.^[^ ^27^ ^]^ We found the expression of Siglec‐G was higher in Tex^term^ than in Tex^prog^ (Figure S4D,E, Supporting Information). In summary, these results indicate that Siglec‐G (Siglec‐10) was highly expressed in TILs, particularly in Tex^term^, and its expression was strongly correlated with coinhibitory receptors in both murine and human.

We then investigated the functional differences between Siglec‐G^+^ and Siglec‐G^−^ intratumor CD8^+^ cells. The production of IFN‐γ, TNF‐α, Granzyme B, perforin, and IL‐2 was reduced in Siglec‐G^+^ cells compared to Siglec‐G^−^ counterpart (Figure 2H–J; Figure S5A,B, Supporting Information). Additionally, the proliferative potential of Siglec‐G^+^ cells was lower (Figure 2K). Furthermore, Siglec‐G^+^ cells had a reduced percentage of CD44^+^CD62L^−^ cells but an increased percentage of CD44^+^CD62L^+^ subset, indicative of a memory CD8^+^ T cell phenotype (Figure S5C, Supporting Information). Collectively, these findings indicate that Siglec‐G expressing CD8^+^ T cells exhibit suppressed function and attenuated proliferation, but enhanced memory potential in tumors.

### 2.3 Siglec‐G^+^ CD8^+^ T Cells Exhibit Reduced Cytotoxicity

**Figure 3:**
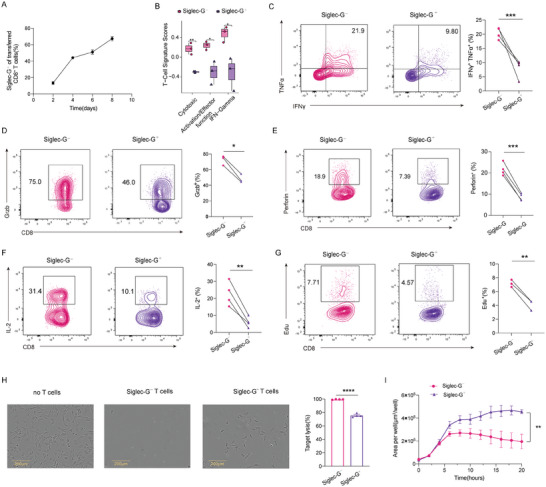
Siglec‐G^−^ CD8+ cells exhibit stronger functional capacities and proliferation potential. A) Representative line chart showing Siglec‐G expression on transferred *Siglecg^Gfp/+^* reporter OT‐I cells after LM‐OVA infection (*n* = 3). B) Gene set variation analysis (GSVA) showing T cell‐associated function pathway from RNA‐seq data in isolated Siglec‐G^−^ and Siglec‐G^+^ OT‐I cells. C–G) Intracellular IFN‐γ and TNF‐α co‐expression (C), Granzyme B (D), Perforin (E), IL‐2 (F) production on Siglec‐G^−^ and Siglec‐G^+^ OT‐I cells and fast proliferative proportion (G) of these two groups (*n* = 3,4). H) Short‐term killing assay showing MC38‐OVA cell lysis after co‐cultured with sorted Siglec‐G^−^ and Siglec‐G^+^ OT‐I cells for 5 hours (E: T ratio, 10:1). I) Long‐term killing assay showing MC38‐OVA cells growth when co‐cultured with sorted Siglec‐G^−^ and Siglec‐G^+^ OT‐I cells over time (E: T ratio, 1:1), both H and I were measured by Incucyte S3 Live Cell Analysis Instrument. Representative data are shown from three independent experiments. The error bar represents mean ± SD. Statistical significance was determined by paired Student's *t*‐test (C–G), unpaired Student's *t*‐test (H), and two‐way ANOVA with multiple comparisons (I). *p < 0.05, **p < 0.01, and ***p < 0.001.

We further characterized Siglec‐G expression in CD8^+^ T cell responses during infection. Recipient mice (CD90.1^+^) were injected of *Siglecg^Gfp/+^* (CD90.2^+^) OT‐I cells and subsequently infected with LM‐OVA. The frequency of Siglec‐G^+^ OT‐I cells progressively increased during infection, predominating the antigen‐specific CD8^+^ T cell population at later time points (**Figure** **3**A). To further investigate whether Siglec‐G^−^ T cells can convert to Siglec‐G^+^ cells and vice versa, we sorted Siglec‐G^−^ and Siglec‐G^+^ OT‐1 cells and transferred them into CD45.1^+^ mice followed by LM‐OVA infection (Figure S6A, Supporting Information). Interestingly, a portion of Siglec‐G^−^ memory T cells expressed Siglec‐G after in vivo activation, whereas some Siglec‐G^+^ memory T cells lost Siglec‐G expression. These results indicate that Siglec‐G^−^ OT‐I cells can shift into Siglec‐G^+^ cells after activation in vivo.

To investigate the functional differences between Siglec‐G^+^ and Siglec‐G^−^ OT‐I effector cells, we sorted these two groups (Figure S6B, Supporting Information) and conducted RNA‐seq analysis. Gene set variation analysis (GSVA) indicated that T cell functions, including cytotoxicity and activation/effector function, along with IFN‐γ, were dysregulated in the Siglec‐G^+^ group (Figure 3B). The production of IFN‐γ, TNF‐α and Granzyme B were reduced in Siglec‐G^+^ OT‐I cells compared to Siglec‐G^−^ counterpart (Figure 3C,D; Figure S6C, Supporting Information). The production of perforin and IL‐2 was also reduced in Siglec‐G^+^ OT‐I cells (Figure 3E,F; Figure S6C, Supporting Information). The proliferative potential of Siglec‐G^+^ OT‐I cells was also impaired during infection (Figure 3G).

To evaluate the cytotoxicity of these two groups, they were incubated with target cells. In a short‐term killing assay, Siglec‐G^−^ OT‐I cells displayed higher killing efficiency, resulting in ≈100% elimination of target cells, compared to Siglec‐G^+^ OT‐I cells, which achieved 80% target cell killing (Figure 3H). Additionally, in the long‐term killing assay, Siglec‐G^−^ OT‐I cells exhibited significantly enhanced inhibition of target cell growth over time (Figure 3I). Therefore, these results collectively demonstrate a reduced killing capacity of Siglec‐G^+^ OT‐I cells.

### 2.4 Siglec‐G Deficiency Promotes Cytotoxicity of CD8^+^ T Cells

**Figure 4:**
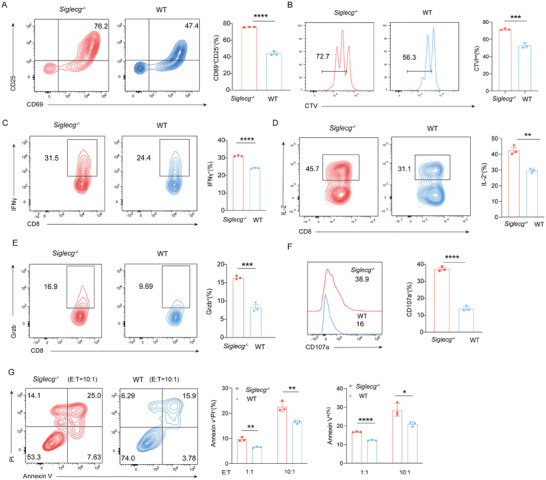
Siglec‐G deficiency enhances CD8^+^ T cell activation, cytokine production, and cytotoxicity in vitro. A) Naïve CD8^+^ OT‐I cells from *Siglecg^−/−^* and WT littermate mice were cultured with BMDCs supplemented with 100 µg mL^−1^ OVA. CD25 and CD69 levels determined by flow cytometry. B) CTV‐labelled naïve CD8^+^ T cells from *Siglecg^−/−^* and WT littermate mice were cultured with BMDCs supplemented with 100 µg mL^−1^ OVA and T cell proliferation (CTV dilution) determined by flow cytometry. C–F) IFN‐γ, IL‐2, Granzyme B and CD107a production on activated CD8^+^ T cells from *Siglecg^−/−^* and WT littermate mice. G) MC38‐OVA cells were labeled by CTV previously and the percentage of Annexin V^+^ PI^+^ and Annexin V^+^ MC38‐OVA cells after co‐cultured with activated *Siglecg^−/−^* and WT OT‐I CD8^+^ T cells for 8 hours. Representative data are shown from three independent experiments. The error bar represents mean ± SD. Statistical significance was determined by unpaired Student's *t*‐test (A–F) and one‐way ANOVA with multiple comparisons (G). *p < 0.05, **p < 0.01, ***p < 0.001, and ****p < 0.0001.

Next, we aimed to elucidate whether Siglec‐G deficiency promotes the function of CD8^+^ T cells. In vitro, Siglec‐G deficiency significantly enhanced the activation of CD8^+^ T cells, as evidenced by the elevated expression of CD69 and CD25 (**Figure** **4**A), and enhanced T cell proliferation (Figure 4B). Additionally, Siglec‐G deficiency promoted the production of IFN‐γ, IL‐2, Granzyme B, and CD107a by CD8^+^ T cells (Figure 4C–F; Figure S7A, Supporting Information). Consistently, deletion of Siglec‐G was associated with enhanced cytotoxicity against tumor cells co‐cultured with CD8^+^ T cells (Figure 4G). Collectively, these results indicate that Siglec‐G deficiency enhanced the activation, function, and cytotoxicity of CD8^+^ T cells in vitro. Similarly, we investigated the effect of Siglec‐G on CD4^+^ T cells and found that Siglec‐G deficiency also promoted the differentiation of Th1 cells (Figure S7B, Supporting Information).

**Figure 5:**
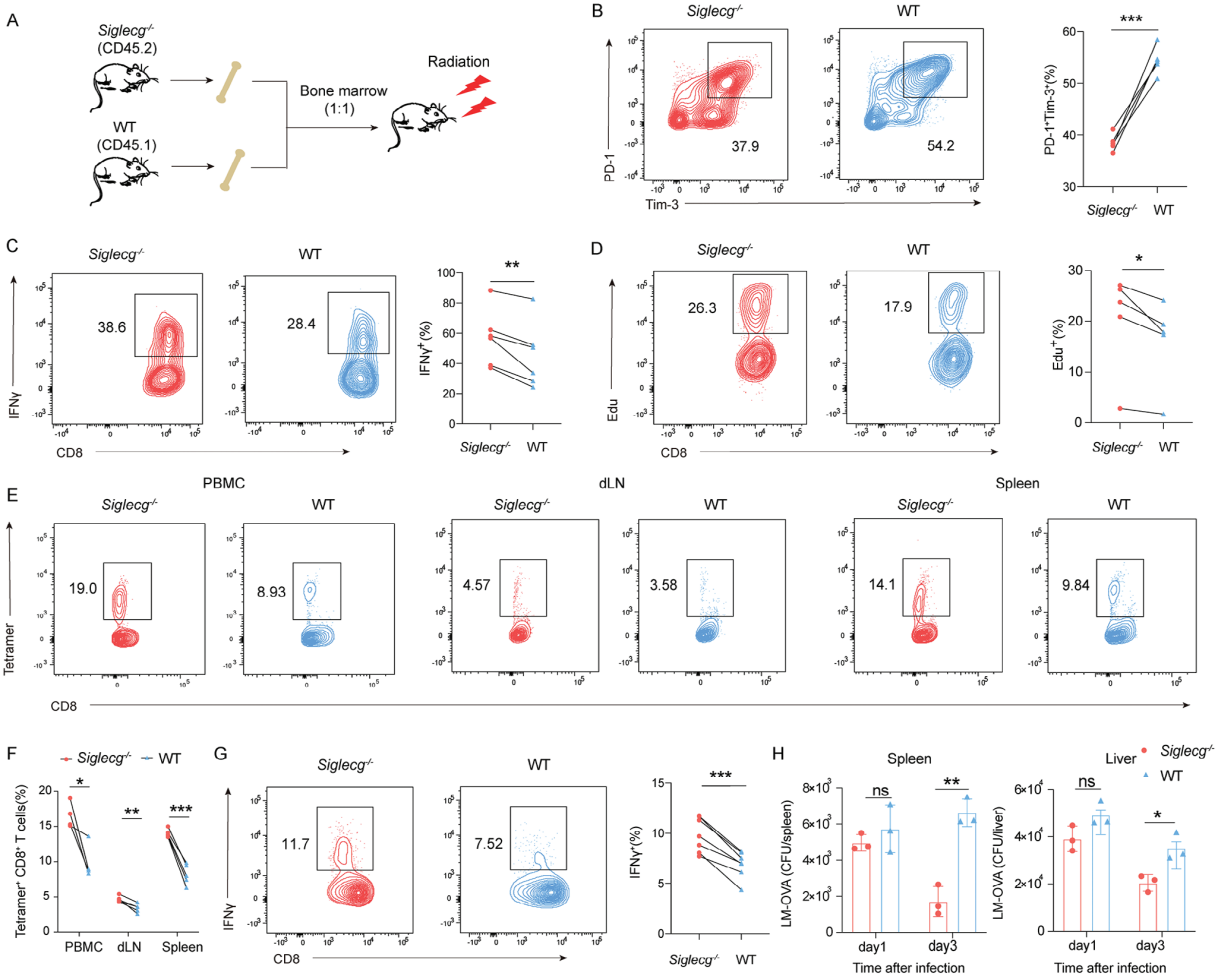
Deletion of Siglec‐G promotes CD8^+^ T cell function and proliferation in vivo. A) The model of bone marrows chimera mice by reconstitution of *Siglecg^−/−^* mice (CD45.2^+^) and WT mice (CD45.1^+^) into recipient mice. B–D) PD‐1 and Tim‐3 positive (B), IFN‐γ producing (C) and Edu positive cells (D) on tumor‐infiltrating CD8^+^ T cells from chimeric mice (*n* = 4–6). E‐G) OVA‐specific tetramer^+^ CD8^+^ T cells in peripheral blood, dLNs, and spleen (E,F), and IFN‐γ‐producing cells (G) from LM‐OVA infected chimera mice (*n* = 4–6). H) CFU in the spleen/liver was determined from *Siglecg* ^−/−^ or WT OT‐I transferred recipient mice which i.v. infected with virulent LM‐OVA (*n* = 3). Representative data are shown from three independent experiments. The error bar represents mean ± SD. Statistical significance was determined by one‐way ANOVA with multiple comparisons (H) and paired Student's *t*‐test (B–G). *p < 0.05, **p < 0.01, and ***p < 0.001.

To further validate whether Siglec‐G deficiency promotes CD8^+^ T cells activity in vivo, we generated bone marrow chimeric mice comprising both WT (CD45.1^+^) and *Siglecg^−/−^* (CD45.2^+^) CD8^+^ T cells (**Figure** **5**A). In this model, the extrinsic environments of WT and *Siglecg^−/−^* CD8^+^ T cells were unified. To investigate the effect of Siglec‐G on T cells in tumors, bone marrow chimeric mice were used to create tumor models and the differences between *Siglecg^−/−^* and WT intratumor CD8^+^ T cells were analyzed. We found that tumor‐infiltrating *Siglecg^−/−^* CD8^+^ T cells expressed lower levels of inhibitory markers such as PD‐1 and Tim‐3 (Figure 5B) and exhibited increased IFN‐γ production compared to their WT counterparts (Figure 5C). Furthermore, *Siglecg^−/−^* CD8^+^ T cells demonstrated higher proliferative potentia (Figure 5D).

In the LM‐OVA infection model using the chimeric mice, we observed a higher frequency of OVA‐specific T cells in *Siglecg^−/−^* CD8^+^ T cells compared to WT counterparts in the peripheral blood, spleen, and dLNs (Figure 5E,F). Additionally, the proliferative potential of *Siglecg^−/−^* CD8^+^ T cells was greater than that of WT cells (Figure S8A, Supporting Information). During the acute infection phase, the proportion of *Siglecg^−/−^* CD8^+^ T cells among the total tetramer‐positive T cells progressively increased, while the proportion of WT CD8^+^ T cells decreased (Figure S8B, Supporting Information). Moreover, the loss of Siglec‐G enhanced IFN‐γ production in CD8^+^ T cells (Figure 5G). Furthermore, *Siglecg* ^−/−^ and WT effector OT‐I cells were respectively transferred into recipient mice, which were then infected with LM‐OVA. On day 3, *Siglecg^−/−^* OT‐I recipient mice exhibited a lower bacterial burden in the spleen and liver (Figure 5H). We also examined the effect of Siglec‐G on memory T cell differentiation. Despite having more effector T cells, *Siglecg^−/−^* CD8^+^ T cells generated fewer memory T cells (Figure S8C, Supporting Information). Taken together, these results indicate the inhibitory role of Siglec‐G in T cells. Siglec‐G deletion enhanced T cells' effector function in both tumor and infection contexts but decreased memory potential during infection. We further compared the *Siglecg^−/−^* CD8^+^ T cells in LM‐OVA infected mice with those *Siglecg^−/−^* CD8^+^ T cells in tumor‐bearing mice. We found a higher exhaustion status of *Siglecg^−/−^* CD8^+^ T cells isolated from tumor‐bearing mice, but higher cytotoxic activity in *Siglecg^−/−^* CD8^+^ T cells isolated from LM‐OVA infected mice (Figure S8F, Supporting Information).

### 2.5 Siglec‐G Impairs CD8^+^ T Cells Function through Metabolic Reprogramming

**Figure 6:**
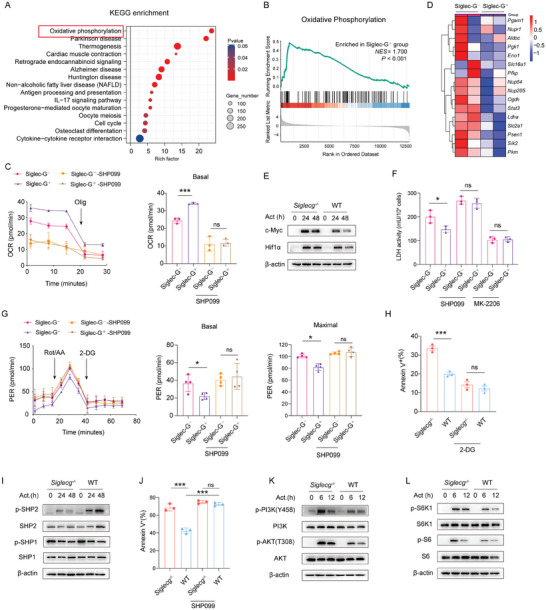
Siglec‐G enhances oxidative phosphorylation of CD8^+^ T cells via SHP‐2. A) KEGG pathway analysis of upregulated genes in Siglec‐G^+^ OT‐I cells compared to Siglec‐G^−^ OT‐I cells by RNA‐Seq (*q*‐value ≤ 0.05, fold change ≥ 2). B) GSEA analysis of OXPHOS‐related genes. C) Seahorse analysis of mitochondrial OCR of isolated Siglec‐G^−^ and Siglec‐G^+^ OT‐I cells, treated with 10 µm SHP2 inhibitor SHP099 for 2 h or not. D) Heatmap of downregulated genes associated with glycolysis in Siglec‐G^−^ CD8^+^ cells relative to that in Siglec‐G^+^ cells. E) Immunoblotting analysis of the indicated proteins in *Siglecg^−/−^* and WT CD8^+^ T cells activated by BMDCs. F) LDH activity in Siglec‐G^+^ and Siglec‐G^−^ OT‐I cells, or treated with 10 µm SHP2 inhibitor SHP099 or AKT inhibitor MK‐2206. G) Seahorse analysis of glycolytic proton efflux rate (glycoPER) of isolated Siglec‐G^−^ and Siglec‐G^+^ OT‐I cells, treated with 10 µm SHP099 or not for 2 h. H) The percentage of Annexin V^+^ MC38‐OVA cells co‐cultured with activated *Siglecg^−/−^* or WT OT‐I CD8^+^ T cells, treated with glycolysis inhibitor 2 mm 2‐DG or not for 4 h (E:T ratio, 10:1). I) Immunoblotting showing phosphorylated and total SHP1 and SHP2 in activated *Siglecg^−/−^* and WT CD8^+^ T cells. J) The percentage of Annexin V^+^ MC38‐OVA cells co‐cultured with activated *Siglecg^−/−^* or WT OT‐I CD8^+^ T cells, treated with SHP099 or not (E:T ratio 10:1). K,L) Immunoblotting analysis of the indicated proteins in *Siglecg^−/−^* and WT CD8^+^ T cells activated as indicated. Representative data are shown from three independent experiments. The error bar represents mean ± SD. Statistical significance was determined by one‐way ANOVA with multiple comparisons. *p < 0.05, ***p < 0.001, and ****p < 0.0001.

To elucidate the mechanisms of Siglec‐G suppression, we reanalyzed the RNA‐seq data of Siglec‐G^+^ and Siglec‐G^−^ OT‐I effector cells. Differential gene enrichment analysis revealed significant enrichment in the OXPHOS pathway in Siglec‐G^+^ CD8^+^ cells (**Figure** **6**A), which was also confirmed by gene set enrichment analysis (GSEA) (Figure 6B). We subsequently measured the oxygen consumption rate (OCR) of Siglec‐G^+^ and Siglec‐G^−^ OT‐I cells. The basal OCR in the Siglec‐G^+^ subgroup was higher than that in the Siglec‐G^−^ counterpart (Figure 6C), confirming Siglec‐G^+^ CD8^+^ effector cells depended more on OXPHOS. Consistently, higher basal OCR and maximal OCR were observed in WT CD8^+^ T cells compared to *Siglecg^−/−^* CD8^+^ T cells (Figure S9A, Supporting Information). Collectively, these results reveal that Siglec‐G could enhance the OXPHOS level of CD8^+^ T cells.

During an acute infection, activated CD8^+^ T cells undergo a metabolic shift from OXPHOS to glycolysis.^[^ ^28^ ^]^ Glycolytic metabolism is essential for CD8^+^ T cells effector function and cytolytic activity.^[^ ^21^, ^29^ ^]^ Increased OXPHOS in Siglec‐G^+^ T cells may be associated with decreased glycolysis, potentially resulting in dampened T cells effector function. We further analyzed our RNA‐seq data and found that the expression of glycolysis‐associated genes, including *Slc2a1, Slc16a1, Ldha, Eno1*, and *Pkm*, were downregulated in Siglec‐G^+^ CD8^+^ cells (Figure 6D). This downregulation was confirmed by qPCR for several glycolysis‐associated genes (Figure S9B, Supporting Information). Notably, the expression of Hif1α and c‐Myc, two transcription factors critical for glycolysis,^[^ ^22^ ^]^ were downregulated in WT CD8^+^ T cells (Figure 6E; Figure S9B, Supporting Information). Lactate dehydrogenase (LDH), a crucial rate‐limiting enzyme in glycolysis that catalyzes the last step of glycolysis,^[^ ^30^ ^]^ also exhibited decreased activity in Siglec‐G^+^ CD8^+^ cells (Figure 6F). Consistently, the activity of LDH (Figure S9C, Supporting Information) and the expression of lactate dehydrogenase A (Ldha) (Figure S9B, Supporting Information) were downregulated in WT T cells. These results further indicate that Siglec‐G reduced glycolysis in CD8^+^ T cells.

Glycolytic proton efflux rate (glycoPER), which excludes additional extracellular acidification from mitochondrial‐derived CO2, serves as a more accurate indicator of glycolytic rates than ECAR. Indeed, Siglec‐G^+^ CD8^+^ cells exhibited significantly lower baseline and maximum PER compared to Siglec‐G^−^ CD8^+^ cells (Figure 6G). Similar results were obtained in WT and *Siglecg^−/−^* CD8^+^ T cells (Figure S9D, Supporting Information). Furthermore, treatment with 2‐DG, a glycolysis inhibitor, abrogated the effect of Siglec‐G on the cytotoxicity of CD8^+^ T cells (Figure 6H). Collectively, these results indicate that Siglec‐G hindered the metabolic transition from OXPHOS to glycolysis in CD8^+^ T cells during activation, ultimately dampening the cytotoxicity of CD8^+^ T cells.

### 2.6 CD24‐Siglec‐G‐SHP2 axis Impairs the Metabolic Switch in Activated CD8^+^ T Cells

We then investigated the signaling mechanisms underlying Siglec‐G‐mediated metabolic transition. The regulatory signaling by most Siglec proteins is attributed to the recruitment of protein tyrosine phosphatases SHP1 or SHP2 to their ITIM and ITIM‐like motifs.^[^ ^31^, ^32^ ^]^ Therefore, we examined whether SHP1 or SHP2 is involved in Siglec‐G‐mediated regulation of T cell activation. Indeed, Siglec‐G enhanced p‐SHP2 but not p‐SHP1 upon CD8^+^ T cell activation (Figure 6I). SHP099, a specific inhibitor of SHP2, significantly reduced the basal OCR of Siglec‐G^+^ cells to the same level as Siglec‐G^−^ CD8^+^ cells (Figure 6C) and eliminated the differences in basal and maximal PER between these two groups (Figure 6G). Similar results were obtained in WT and *Siglecg^−/−^* CD8^+^ T cells (Figure S9A,D, Supporting Information). SHP099 increased the LDHA activity in Siglec‐G^+^ cells to the same level as Siglec‐G^−^ CD8^+^ cells (Figure 6F). Furthermore, SHP099 also abrogated the differences in activation/proliferation (Figure S10A, Supporting Information) and cytotoxicity between WT and *Siglecg* ^−/−^ T cells (Figure 6J). Thus, these results collectively demonstrate that Siglec‐G increased OXPHOS and decreased glycolysis mainly via SHP2 signaling in CD8^+^ T cells, thereby impairing CD8^+^ T cells' function. Additionally, slightly decreased phosphorylation of NF‐κB and STAT3 was detected in activated WT CD8^+^ T cells compared with *Siglecg^−/−^* T cells (Figure S10B, Supporting Information), indicating that these may be downstream effectors of Siglec‐G/SHP2.^[^ ^33^ ^]^

During acute infection, the metabolic shift from OXPHOS to glycolysis in CD8^+^ T cells is mediated by the PI3K‐AKT pathway.^[^ ^28^ ^]^ It has been reported that SHP2 can inhibit downstream PI3K‐AKT signaling.^[^ ^34^, ^35^ ^]^ Immunoblot analysis revealed decreased phosphorylation of PI3K and AKT in activated WT CD8^+^ T cells compared with *Siglecg^−/−^* cells (Figure 6K). MK‐2206, a specific inhibitor of AKT, abrogated the differences in LDH activity between Siglec‐G^+^ and Siglec‐G^−^ CD8^+^ cells (Figure 6F). Similar results were obtained in WT and *Siglecg^−/−^* CD8^+^ T cells (Figure S9C, Supporting Information). Furthermore, MK‐2206 also abrogated differences in cell activation, proliferation (Figure S10A, Supporting Information), and cytotoxicity (Figure S10C, Supporting Information) between WT and *Siglecg^−/−^* CD8^+^ T cells. Taken together, these results indicate the essential role of PI3K‐AKT signaling in Siglec‐G‐mediated suppression in CD8^+^ T cells.

As PI3K‐AKT signaling activates mTOR,^[^ ^36^, ^37^ ^]^ and mTOR regulates glycolysis by promoting the expression of Hif1α and c‐Myc,^[^ ^22^, ^38^ ^]^ we next examined the activity of mTORC1 in CD8^+^ T cells. Immunoblot analysis revealed decreased phosphorylation of ribosomal protein S6K1 and ribosomal protein S6 in WT CD8^+^ T cells (Figure 6L), indicative of reduced mTORC1 activation. Rapamycin, a specific inhibitor of mTOR, abrogated the differences in cytotoxicity between WT and *Siglecg^−/−^* CD8^+^ T cells (Figure S10D, Supporting Information). Collectively, these results suggest that Siglec‐G exerted its function through PI3K‐AKT‐mTOR signaling in CD8^+^ T cells.

The effect of SHP2 and AKT in Siglec‐G mediated T cell suppression was also examined in vivo. An equal proportion of *Siglecg* ^−/−^ (CD45.2^+^) and WT (CD45.1^+^) OT‐I cells, with or without SHP2 knockdown, were injected into recipient mice (CD90.1^+^) that had been infected with LM‐OVA (Figure S10E, Supporting Information). Six days later, an increased proportion of *Siglecg* ^−/−^ T cells compared to WT cells were found in the spleen. However, this disparity was abrogated when SHP2 was knocked down (Figure S10F, Supporting Information). Similarly, the knockdown of SHP2 also eliminated the differences in proliferation and cytotoxicity between *Siglecg* ^−/−^ and WT OT‐I cells in vivo (Figure S10G,H, Supporting Information), confirming the essential role of SHP2 in Siglec‐G mediated T cell suppression. In addition, the cytotoxicity of *Siglecg* ^−/−^ and WT OT‐I cells on day 6 was compared in the presence or absence of the SHP2 inhibitor SHP099, the AKT inhibitor MK‐2206, or 2‐DG. SHP099 increased, while MK‐2206 and 2‐DG attenuated the cytotoxicity of T cells, and all three treatments abrogated the differences between WT and *Siglecg* ^−/−^ T cells (Figure S10I,J, Supporting Information). Taken together, these findings further confirm the effect of SHP2, AKT, and glycolysis in Siglec‐G‐mediated T cell suppression in vivo.

It is notable that CD24, a heavily glycosylated glycosylphosphatidylinositol‐anchored protein widely expressed on solid tumors, including MC38 cells, has been identified as a ligand for Siglec‐G.^[^ ^39^, ^40^ ^]^ We further investigated whether CD24 was involved in the suppression effects of Siglec‐G on T cells. During tumor‐killing assays, anti‐CD24 neutralizing antibodies significantly rescued the Siglec‐G‐mediated inhibition of CD8^+^ T cells cytotoxicity (Figure S10K, Supporting Information). Similarly, anti‐CD24 neutralizing antibodies significantly inhibited SHP2 activation, thereby promoted the activation of PI3K‐AKT‐mTOR signaling (Figure S10L,M, Supporting Information). Furthermore, removing sialic acids from tumor cell surfaces using sialidase (also named neuraminidases) enhanced the cytotoxic CD8^+^ T cell‐mediated killing of tumor cells in vitro (Figure S10N, Supporting Information). Collectively, these data suggest that the interaction between CD24 and Siglec‐G impaired the metabolic shift from OXPHOS to glycolysis in CD8^+^ T cells through the SHP2‐PI3K‐AKT‐mTOR pathway.

### 2.7 Siglec‐G Deletion in CD8^+^ T Cells Improves Anti‐Tumor Immunity

**Figure 7:**
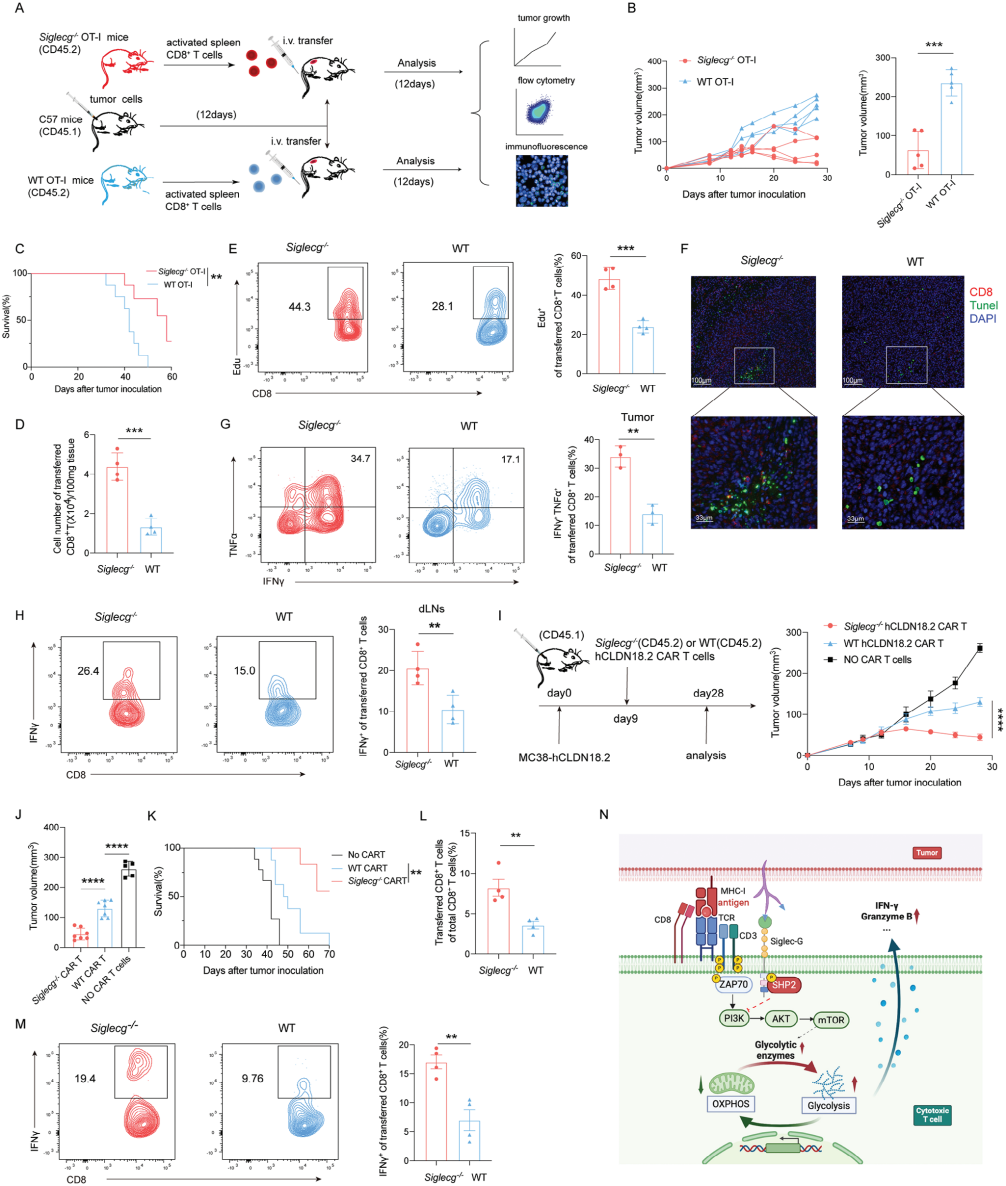
Siglec‐G deletion in CD8^+^ T cells enhances anti‐tumor immunity. A) CD8^+^ T cells from the spleen of *Siglecg* ^−/−^ and WT OT‐I littermate mice (CD45.2^+^) were activated as indicated and transferred to tumor‐bearing mice (CD45.1^+^). Intratumor‐transferred OT‐I CD8^+^ T cells were analyzed 12 days later. B) Tumor growth of MC38‐OVA‐bearing mice were measured every 2–3 days and tumor were dissected at last day (*n* = 5). C) Survival analysis of MC38‐OVA‐bearing mice. D) The proportions of adoptively transferred *Siglecg* ^−/−^ and WT OT‐I CD8^+^ T cells in total intratumor CD8^+^ T cells (*n *= 4). E) Quantification of Edu positive cells in intratumor adoptively transferred *Siglecg* ^−/−^ and WT OT‐I CD8^+^ T cells (*n* = 4). F) Representative immunofluorescence staining of CD8 (red) or Tunel (green) in tumor. Scale bar, 100 µm (magnified: 33 µm). G,H) IFN‐γ and TNF‐α expression in adoptively transferred *Siglecg* ^−/−^ and WT OT‐I CD8^+^ T cells from tumors (G) and dLNs (H) (*n *= 4). I,J) MC38‐hCLDN18.2 tumor cells were injected s.c. into CD45.1^+^ mice followed by the adoptive transfer of 2 × 10^6^ CAR T cells generated from *Siglecg* ^−/−^ and WT splenocytes (CD45.2^+^). Tumor growth of MC38‐hCLDN18.2‐bearing mice were measured every 2–3 days and tumor were dissected at last day (*n* = 7). K) Survival analysis of MC38‐hCLDN18.2‐bearing mice. L) The proportions of adoptively transferred *Siglecg* ^−/−^ and WT CAR T cells in total intratumor CD8^+^ T cells (*n* = 4). M) IFN‐γ expression in adoptively transferred *Siglecg* ^−/−^ and WT CAR T cells tumor infiltrates (*n* = 4). N) The mechanism of Siglec‐G suppressing CTL responses through SHP2‐PI3K‐AKT‐mTOR mediated metabolic rewiring. Representative data are shown from three independent experiments. The error bar represents mean ± SD. Statistical significance was determined by two‐way ANOVA with multiple comparisons (B and I), a Log‐rank (Mantel–Cox) test (C and K), and unpaired Student's *t‐*test (D, E, G,H, J, L, and M). **p < 0.01, ***p < 0.001, and ****p < 0.0001.

We next investigated whether monitoring Siglec‐G would enhance the therapeutic effect of adoptive T cell transfer in tumors. Activated WT and Siglec‐G deficient OT‐I CD8^+^ T cells (CD45.2^+^) were transferred into tumor‐bearing mice (CD45.1^+^) respectively (**Figure** **7**A). The genetic ablation of Siglec‐G in adoptively transferred CD8^+^ T cells greatly inhibited the growth of MC38‐OVA tumors (Figure 7B; Figure S11A, Supporting Information) and significantly increased the survival of mice (Figure 7C). Further analysis showed that the infiltration and proliferative potential of intratumor OT‐I CD8^+^ T cells were significantly increased in the absence of Siglec‐G (Figures 7D,E). Immunohistochemical analysis of CD8^+^ T cells and apoptotic tumor cells revealed that Siglec‐G deficiency promoted the aggregation of CD8^+^ T cells and the apoptosis of tumor cells (Figure 7F). Similarly, the loss of Siglec‐G enhanced IFN‐γ, TNF‐α, Granzyme B, perforin, and IL‐2 production in adoptively transferred OT‐I CD8^+^ T cells from both tumors and dLNs (Figure 7G,H; Figure S11B–D, Supporting Information). Consistent results were obtained in B16‐OVA‐bearing mice (Figure S11E–I, Supporting Information). Siglec‐G deficiency also led to decreased PD‐1 and Tim‐3 expression on adoptively transferred CD8^+^ T cells (Figure S11J, Supporting Information).

To elucidate whether Siglec‐G depletion synergizes with anti‐PD‐1 antibody in tumor immunotherapy, MC38‐OVA bearing mice were treated with activated *Siglecg^−/−^* and WT CD8^+^ T cells plus α‐PD‐1 every three days. The addition of α‐PD‐1 further enhanced tumor growth inhibition (Figure S12A,B, Supporting Information). Moreover, α‐PD‐1 treatment decreased the expression of Siglec‐G (Figure S12C, Supporting Information). Collectively, these results indicate that targeting Siglec‐G may improve the therapeutic effect of adoptive T cell therapy and has a synergistic effect with anti‐PD‐1 therapy.

### 2.8 Targeting Siglec‐G Enhances CAR T Cell Anti‐Tumor Immunity

Chimeric antigen receptor (CAR) T cell therapy has emerged as a revolutionary immunotherapy strategy in cancer treatment.^[^ ^41^ ^]^ We then examined whether targeting Siglec‐G would improve the efficacy of adoptively transferred CAR T cells in solid tumors. To this end, human Claudin18.2 (hCLDN18.2)‐targeted CAR T cells were utilized.^[^ ^42^ ^]^ The deletion of Siglec‐G did not influence the generation of CAR T cells (Figure S12D,E, Supporting Information) but significantly enhanced their capacity to specifically kill hCLDN18.2‐expressing MC38 tumor cells in vitro (Figure S12F, Supporting Information).

To assess the impact of Siglec‐G deletion on the therapeutic efficacy of CAR T cells in vivo, we adoptively transferred Siglec‐G‐deficient and WT α‐hCLDN18.2 CAR T cells into mice bearing hCLDN18.2‐expressing MC38 tumors respectively. Strikingly, the adoptive transfer of Siglec‐G‐deficient α‐hCLDN18.2 CAR T cells resulted in more significant repression of tumor growth compared to WT CAR‐T cells (Figure 7I,J; Figure S12G, Supporting Information), and considerably extended the survival of mice (Figure 7K). This was accompanied by a substantial increase in the number of Siglec‐G‐deficient CAR T cells in the tumor (Figure 7L). Moreover, intratumor Siglec‐G‐deficient CAR T cells exhibited improved cytotoxicity, as indicated by elevated IFN‐γ expression (Figure 7M). In summary, our study identify Siglec‐G as an inhibitory receptor on T cells that suppressed T cell function through SHP2‐regulated metabolic reprogramming and can be targeted in adoptive T cell therapy for tumors (Figure 7N).

## 3 Discussion

CD8^+^ T cells are a vital component of the adaptive immune response and play a crucial role in antitumor immunity. Recent studies have revealed that Siglecs family genes can regulate the interaction between immune cells and tumor cells by sialic acid‐dependent mechanisms, indicating that these Siglecs could be potential targets in checkpoint therapy.^[^ ^43^, ^44^, ^45^ ^]^ In this study we demonstrate that the expression of Siglec‐G was significantly increased on both human and murine CD8^+^ and CD4^+^ TILs. The CD24‐Siglec‐G‐SHP2 axis was critical for coordinating PI3K‐AKT signaling and metabolic reprogramming in T cells, thereby affecting their activation, expansion, and cytotoxic activity. Thus, Siglec‐G may serve as an inhibitory receptor on T cells, similar to PD‐1, Tim‐3, and TIGIT. Importantly, we report that targeting Siglec‐G can enhance the antitumor immunity of T cells and improve the efficacy of adoptively transferred CAR T cells.

Sialoglycans are frequently overexpressed in cancer cells,^[^ ^10^ ^]^ and their receptors are widely expressed on various immune cells.^[^ ^31^ ^]^ The interplay between Siglec receptors and sialoglycans profoundly affects immune cell function, contributing to the establishment of an immunosuppressive microenvironment.^[^ ^46^, ^47^ ^]^ Siglec‐15 on macrophages has been shown to inhibit T cell‐mediated antitumor immunity.^[^ ^44^ ^]^ Several studies have revealed that Siglec‐7 and Siglec‐9 on NK cells can be engaged by cancer‐associated sialoglycans to inhibit antitumor immune activation.^[^ ^12^, ^48^ ^]^ Siglec‐7 and Siglec‐9 have also been demonstrated to induce a pro‐tumorigenic macrophage phenotype by engaging sialoglycans on pancreatic adenocarcinoma cells.^[^ ^49^ ^]^ In addition, Siglec‐9 has been found to be upregulated on tumor‐infiltrating T cells, and reduction of Siglec‐9 ligands on tumor cells significantly induces T cell activation and tumor cell killing.^[^ ^43^, ^50^ ^]^ As for Siglec‐G (Siglec‐10), the interaction between Siglec‐10 on tumor‐associated macrophages (TAMs) and sialylated CD24 on cancer cells can polarize TAM into cancer‐supporting pro‐tumorigenic M2‐like macrophages and inhibit phagocytosis.^[^ ^17^ ^]^ Furthermore, Siglec‐G can suppress in vitro and in vivo T cell responses in the presence of certain DAMPs, mitigating GVHD or autoinflammatory response.^[^ ^51^, ^52^ ^]^ Here, we found that Siglec‐G(Siglec‐10) was primarily expressed on exhausted CD8^+^ T cells in tumors and can suppress T cell function. We have also confirmed CD24 on cancer cells as a sialoglycan ligand that interacts with Siglec‐G on T cells. Due to the complexity and diversity of sialic acids, it is possible that other ligands of Siglec‐G, such as CD52, may also interact with Siglec‐G on T cells.^[^ ^52^ ^]^ Furthermore, treatment of tumor cells with sialidase resulted in a slightly enhanced killing ability of *Siglecg* ^−/−^ T cells, Therefore, Siglec‐9 or Siglec‐15 may have redundant roles with Siglec‐G, warranting further investigation.

Our results indicate that memory T cells from naïve mice expressed higher levels of Siglec‐G, and antigen‐specific Tcm and Tem isolated from LM‐OVA infected *Siglecg^Gfp/+^* mice also exhibited higher Siglec‐G expression. Consistently, Siglec‐G deficiency in T cells resulted in increased effector T cells, but reduced memory T cells in WT and *Siglecg^−/−^* chimeric mice. Collectively, these data may indicate that Siglec‐G^+^ cells showed enhanced memory potential and superior capacity to persist long‐term in vivo. PD‐1 is a well‐characterized marker of exhausted T cells. It has been reported that the genetic deletion of PD‐1 enhances early effector CD8^+^ T cell responses but results in subsequently compromised memory CD8^+^ T cells.^[^ ^53^ ^]^ PD‐1 expression on antigen‐specific CD8^+^ T cells is required for the development of a durable CD8 T cell memory pool.^[^ ^54^ ^]^ Thus, the discriminated role of Siglec‐G in effector T cells, exhausted T cells, and memory T cells mirrors the effect of PD‐1 in these different T cells. The precise mechanism through which Siglec‐G influences the generation or maintenance of memory‐T‐cells warrants further exploration. We hypothesized that key molecules or pathways involved in CD8^+^T‐cell‐memory might be regulated by Siglec‐G. Additionally, the reprogramming of CD8 T cells from cytotoxic effector to quiescent memory state is associated with a metabolic shift to decreased glycolysis and increased fatty acid oxidation.^[^ ^55^ ^]^ PD‐1 promotes long‐term memory homeostasis through inhibition of mTOR‐dependent glucose oxidation and enhancement of fatty acid oxidation.^[^ ^54^ ^]^ Considering the implication of Siglec‐G in the regulation of glycolytic metabolism, the reduced memory T‐cells in Siglec‐G deficient cells may be related to metabolic regulation.

During the priming phase, in the absence of tumor cells, Siglec‐G can also inhibit activation, function, and cytotoxicity of CD8^+^ T cells, particularly in the context of BMDC co‐culture. It is probably because sialoglycan‐ligands are expressed not only on tumors. CD24 can also be expressed at the surface of most immune cells, including dendritic cells and T cells.^[^ ^56^, ^57^ ^]^ For example, a DC subset expressing CD24 in early metastases can expand Tregs and suppress CD8^+^ T cells, establishing an immunosuppressive microenvironment conducive to metastasis formation.^[^ ^58^ ^]^ In addition, CD24‐expressing donor T cells negatively regulate DAMP‐dependent allogeneic responses.^[^ ^59^ ^]^ Therefore, Siglec‐G on T cells can be activated by CD24 expressed on DC or T cells during in vitro activation.

As one of the most studied immune checkpoint receptors, PD‐1 can recruited SHP1/SHP2 to its ITIM and ITIM‐like motif, thereby antagonizing signals from TCR and CD28.^[^ ^34^, ^35^ ^]^ Similarly, Siglec‐G inhibited PI3K‐AKT‐mTOR pathway through the recruitment of SHP2. Despite the partial mechanistic similarity between Siglec‐G and PD‐1 in mediating T cell inhibition, their expression patterns did not fully overlap, indicating complementary functions of these checkpoints in exhausted cells. This may also explain the synergistic effect of Siglec‐G depletion and anti‐PD‐1 antibody in tumor immunotherapy. Moreover, CTLA4 can also interact with SHP2 to interfere with TCR signaling.^[^ ^60^ ^]^ Tim‐3, upon ligand interaction, leads to intracellular domain phosphorylated, the release of BAT3, and the recruitment of FYN, which inactivates T cells.^[^ ^61^ ^]^ LAG‐3 activation causes the dissociation of LCK from the CD4 or CD8 co‐receptor, leading to a higher threshold for TCR activation.^[^ ^62^ ^]^ Recently, three papers elaborate that combined deficiency of LAG‐3 and PD‐1 in CD8^+^ T cells increased proximal TCR signaling, leading to increased proliferation and cytokine production.^[^ ^63^, ^64^, ^65^ ^]^ Since the mechanisms of Siglec‐G are different from those of Tim‐3 or LAG3, we hypothesize that combined suppression of Siglec‐G and Tim‐3 or LAG‐3 may also potentiate a robust effector CD8^+^ T cell program through non‐redundant‐mechanisms

The PI3K/AKT signaling pathway downstream of SHP2 can modulate T cell metabolism, especially glycolysis, which is important for CD8^+^ T cell effector function.^[^ ^21^, ^29^ ^]^ As reported, PD‐1 and CTLA‐4 also inhibit glycolysis, thereby inhibiting T cell cytotoxicity and anti‐tumor immunity.^[^ ^66^ ^]^ In addition, the fatty acid β‐oxidation (FAO) is increased in the presence of PD‐1 but not CTLA‐4.^[^ ^66^ ^]^ Our data indicated that Siglec‐G impaired glycolysis in CD8^+^ T cells, but whether Siglec‐G affects FAO needs further investigation. Tumor cells primarily rely on aerobic glycolysis for energy production, even under oxygen‐sufficient conditions. The competition for glucose between tumors and T cells may modulate the function of immune cells and influence cancer progression.^[^ ^67^ ^]^ Combined therapy with PD‐1 and CTLA4 promoted T cell glycolysis, thereby restricting tumor cells’ access to enough glucose.^[^ ^67^ ^]^ Thus, the synergistic effect of Siglec‐G and PD‐1 may further enhance glycolysis in T cells, altering the metabolic pressure in the nutrient‐deficient TME. Moreover, a recent study demonstrated that PD‐1 signaling promotes the production of unsaturated phospholipids and stimulates ferroptosis.^[^ ^68^ ^]^ It's interesting to consider whether phospholipids and other metabolites are involved in T cell inhibition by Siglec‐G.

In our study, Siglec‐G is upregulated in exhausted T cells, particularly Tex^term^, and contributes to the exhaustion and functional inhibition of CD8^+^ T cells. Tox has been identified as a transcription factor that contribute to T cell exhaustion.^[^ ^69^ ^]^ LAG‐3 has been reported to regulate the expression of TOX.^[^ ^63^ ^]^ Therefore, Siglec‐G might also modulate the expression of TOX in exhausted T cells. Furthermore, considering the reliance of T cell function on metabolic reprogramming, additional research is warranted to investigate the correlation between metabolism and exhaustion of Siglec‐G^+^ CD8^+^ cells within the TME.

In addition to the potential to enhance endogenous anti‐tumor immunity and anti‐bacteria activity, our study also demonstrated the therapeutic effect of targeting Siglec‐G in adoptive cell therapy and CAR‐T therapy. CAR T therapy has been confirmed effective in treating hematological malignancies, but is application in solid tumors remains challenging.^[^ ^70^ ^]^ Innovative strategies to overcome the limitations of CAR‐T cell therapy have been considered, such as targeting multiple antigens, engineering CAR structure to express chemokine receptors and combination with checkpoint blockade.^[^ ^71^ ^]^ Herein, Siglec‐G deleted CAR T may overcome exhaustion intrinsically, consistent with the effect of PD‐1 knockout CAR T.^[^ ^72^, ^73^ ^]^ Further studies exploring the possibilities of targeting Siglec‐G‐associated therapy are necessary. For instance, CAR T cells can be engineered to secret Siglec‐G neutralizing antibodies, which can not only improve the anti‐tumor activity of T cells but also induce macrophage‐dependent phagocytosis. Additionally, combining Siglec‐G targeted CAR‐T therapy with checkpoint blockade may also be more effective.

Finally, as the GFP coding sequence replaced the Siglec‐G coding gene in *Siglecg^Gfp/+^* mice, its expression is controlled by the regulatory elements of the Siglec‐G promoter. Consequently, GFP expression can serve as an indicator of Siglec‐G transcription.^[^ ^74^ ^]^ However, it should be noted that the presence of GFP cannot distinguish between membrane and cytoplasmic Siglec‐G. In addition, the differences in translocation and degradation between Siglec‐G and GFP may influence the efficacy of using GFP as a surrogate marker for membrane Siglec‐G. Therefore, while GFP expression partially reflects surface Siglec‐G expression, GFP^+^ T cells and Siglec‐G^+^ T cells are not completely the same.

In summary, our findings illustrate the crucial role of Siglec‐G in inhibiting activation, expansion, and cytotoxicity of CD8^+^ T cells. Siglec‐G, mediated by SHP2, impaired the metabolic reprogramming from OXPHOS to glycolysis in CD8^+^ T cells. Our study reveals that Siglec‐G may function as an inhibitory immune checkpoint, and targeting Siglec‐G could be a promising therapeutic strategy to enhance antitumor immunity and remodel metabolism in TME.

## 4 Experimental Section

### Mice

*Siglecg^Gfp/+^* GFP Knockin mice were kindly provided by Yang Liu.^[^ ^74^ ^]^ Briefly, a GFP coding sequence with a stop code was used to replace the *Siglecg* coding gene. The *Siglecg^Gfp/Gfp^* mice lacking SiglecG expression were used as *Siglecg^−/−^ mice*. Litterate *Siglecg^Gfp/+^* and *Siglecg^+/+^* mice were used as control. The heterozygous *Siglecg^Gfp/+^* mice were used as reporter mice. OT‐I (C57BL/6‐Tg (TcraTcrb)1100Mjb/J, 003831), CD45.1 (B6.SJL‐PtprcaPepcb, 002014) and Thy1.1 (B6.PL‐Thy1a/CyJ, 000406) mice were obtained from The Jackson Laboratory. C57BL/6 mice were purchased from Joint Ventures Sipper BK Experimental Animal Company (Shanghai, China). *Siglecg^−/−^* OT‐I mice and *Siglecg^+/−^* OT‐I mice were generated by breeding. All mice were bred in specific‐pathogen‐free conditions. All animal experiments were undertaken in accordance with the National Institute of Health Guide for the Care and Use of Laboratory Animals with the approval of the Scientific Investigation Board of Naval Medical University, Shanghai.

### Cells and Cell Culture

All cell lines were cultured under an atmosphere of 5% CO_2_ at 37 °C. MC38 and MC38‐OVA cells which were cultured in a DMEM medium containing 10% FBS. B16 cells were cultured in a DMEM‐F12 medium containing 10% FBS. MC38‐hCLDN18.2 cells purchased from Sanyou Bio were cultured in DMEM medium containing 10% FBS and 3 µg ml^−1^ blasticidin.

### Human Blood Specimens and Colorectal Cancer (CRC) Tissue Specimens

Written informed consent was obtained from each patient with cancer and a healthy volunteer. The protocols for human specimen collection were approved by the Institutional Review Boards of Shanghai Changhai Hospital, Navy Medical University under approval number B2022‐204 and performed in accordance with the procedures and guidelines of the Human Research Ethics Committee.

### Analysis of Public scRNA‐Seq Data

Public scRNA‐seq data for tumor‐infiltrating CD8^+^ T cells from human colorectal cancer were obtained from GSE231559.^[^ ^75^ ^]^ The Seurat package was used to normalize data, dimensionality reduction, clustering, and differential expression. Seurat alignment method canonical correlation analysis was used for integrated analysis of data sets. For clustering, highly variable genes were selected, and the principal components based on those genes were used to build a graph, which was segmented with a resolution of 0.4.

### RNA Sequencing

Total RNA was extracted from isolated cells using TRIzol reagent (Invitrogen) and was subjected to librarying and high‐throughput sequencing using the Hiseq platform (Illumina). Sequencing raw reads were filtered and trimmed by Seqtk (https://github.com/lh3/seqtk), and then mapped by mouse genome mm10 using Hisat2(version:2.0.4). Gene expression levels were calculated using StringTie. Differentially expressed genes were analyzed by edgeR, and expression variations with both *q*‐value ≤ 0.05 and fold‐change ≥ 2 were considered significant. For the sequence of Siglec‐G+ and Sigelc‐G‐ OT‐I cells, raw data were deposited in gene expression omnibus (GEO) under accession number: GSE255336. For the sequence of Tex in tumor‐bearing mice and Teff in LM‐OVA infection mice, raw data were deposited in GEO under accession number: GSE272544.

In this study, GSEA (Gene set enrichment analysis) was conducted to capture significantly enriched pathways to analyze the underlying molecular mechanisms. The enrichment and visualization process were carried out following previous descriptions.^[^ ^76^ ^]^ GSVA (Gene Set Variation Analysis) was applied to investigate the effect of Siglec‐G on the functional effects (activation, secretion, and cytotoxicity) of CD8^+^ T cells. The samples in the normalized expression matrix were scored for functional enrichment based on the R package “GSVA”, and the differences in scores between the two groups were then compared. The involved gene sets were obtained from publicly available databases or published literature (Table S1, Supporting Information).

### Tumor‐Bearing Mouse Models

MC38, MC38‐OVA, MC38‐hCLDN18.2, or B16 cells (1 × 10^6^) were transplanted subcutaneously into the right flank of mice. Tumor length and width were measured with a digital caliper every 2–3 days to calculate tumor volume (=0.5 × *length × width* ^2^). For analysis of tumor‐infiltrating lymphocytes, mice were sacrificed when the tumors reached 500 mm^3^, and the tumors were resected for subsequent analysis.

### Preparation of Single‐Cell Suspensions

The tumor tissue from tumor‐bearing mice or CRC patients was mechanically dissociated into smaller pieces, and digested in DMEM medium supplemented with 1 mg mL^−1^ collagenase type IV and 200 µg mL^−1^ DNase I at 37 °C for 60 min, and then passed through a 40 µm cell strainer. Similarly, dLNs and spleens were digested as described above for 15 or 30 min, before passing through 40 µm cell strainers.

### Flow Cytometry and Intracellular Cytokine Staining

The following antibodies against human were used for flow cytometry: BV421‐CD45 (BioLegend Cat#368521); BUV737‐CD3(BD Biosciences Cat#612752); PE‐cy7‐CD4(BD Biosciences Cat#560649); AF700‐CD8(BD Biosciences Cat#344723); BUV395‐CD8(BD Biosciences Cat#563795); PE‐Siglec‐10(BioLegend Cat#347603); FITC‐PD‐1(BioLegend Cat#379205); BV421‐CCR7(BioLegend Cat#353207); APC‐CD45RA (BioLegend Cat#304111); FITC‐CD45RO (BioLegend Cat#304204)

Antibodies to mouse proteins were as follows: BD Horizon Fixable Viability Stain 510 (BD Biosciences Cat#564406); PerCP‐Cy5.5‐CD45.2 (BioLegend Cat#109828); APC‐Cy7‐CD45.1 (BioLegend Cat#110716); APC‐CD90.2 (BioLegend Cat#105312); PE‐cy7‐CD3 (BD Biosciences Cat#552772); APC‐CD8a (BioLegend Cat#100712); BV421‐CD8a (BioLegend Cat#100738); PE‐cy7‐CD4 (BioLegend Cat#100421); PerCP‐Cy5.5‐CD4 (BioLegend Cat# 100433); BV421‐PD‐1 (BioLegend Cat#135218); PE‐Tim‐3 (BioLegend Cat#119703); APC‐TCR Va2 (BioLegend Cat#127810); APC‐IFNγ (BioLegend Cat#505809); PE‐granzyme B (BioLegend Cat#372207); PE‐CD69 (BioLegend Cat#104507); APC‐CD25 (BioLegend Cat#101909); PE‐CD107a (BioLegend Cat#144011); PE‐cy7‐B220 (BD Biosciences Cat#552772); PE‐CD44 (BioLegend Cat# 103023); APC‐CD62L (BioLegend Cat# 104412); APC‐Ly108 (BioLegend Cat#134609); BV421‐IL2 (BioLegend Cat#503825); PE‐cy7‐TNF‐α (BioLegend Cat#506323); APC‐Perforin (BioLegend Cat#154303); eFluor 660‐TOX (eBioscience Cat#50‐6502‐80); APC‐Siglec‐G (eBioscience Cat#17‐5833‐82); H‐2K^b^ OVA Tetramer‐SIINFEKL‐PE (MBL Life science code No. TS‐5001‐1C). Single cell suspensions were prepared from the indicated tissues and subjected to flow cytometry using SONY ID7000 or LSR Fortessa (BD Biosciences). Flow cytometry data were analyzed by FlowJo software. Single cell suspensions were stained with antibodies against surface molecules at 4 °C for 30 min after incubation with anti‐CD16/CD32 for 5 min to block non‐specific antibody binding. For intracellular cytokine staining, cells were stimulated with a cell stimulation cocktail plus protein transport inhibitor (BioLegend) for 10 h prior to staining with antibodies against surface proteins followed by fixation and permeabilization with the BD Cytofix/Cytoperm kit and then staining with antibodies against intracellular antigens. Fluorescence minus one control combined with isotype control was used as a negative control for intracellular staining. Cell‐Light EdU Apollo488 In Vitro Kit was used to detect intracellular EdU in T cells according to the manufacturer's instructions. The APC Annexin V Apoptosis Detection Kit II from BD Biosciences was used to detect apoptotic tumor cells according to the manufacturer's instructions.

### In Vitro Murine CD8^+^ T Cells Experiments

CD8^+^ T cells used for in vitro experiments were isolated from the spleen of wild type and *Siglecg^−/−^* mice using EasySep Mouse Naïve CD8^+^ T cell isolation Kit (STEMCELL Technologies) according to product protocol. Primary cells were cultured in plates coated with 5 µg mL^−1^ anti‐CD3 (BD Biosciences) and 1 µg mL^−1^ anti‐CD28 (BD Biosciences) in complete RPMI 1640 medium as described before. In some cases, Naïve CD8^+^ OT‐I cells were cultured with Bone marrow‐derived DCs (BMDCs) in a complete RPMI 1640 medium supplemented with 100 µg mL^−1^ OVA. For CD8^+^ T cell activation evaluation, cells were harvested at 48‐hour post‐stimulation and stained against surface markers (such as CD25 and CD69) for flow cytometry analysis. For CD8^+^ T cells function analysis, cells were harvested at 48‐hour post‐stimulation and treated with protein transport inhibitor for 10 h prior to further flow cytometry staining as previously described.

### In Vitro Human CD8^+^ T Cells Experiments

PBMCs were enriched from whole blood of CRC patients and volunteers by density gradient cell separation. Briefly, blood was collected in medical EDTA tubes and diluted to a 1:1 volume ratio with the D‐PBS. Then, the mononuclear cells were purified by Ficoll‐Paque PREMIUM density gradient media (cytiva). After counted, the PBMCs were ready for further experiments. For the CD8^+^ T cells activation experiment, human CD8^+^ T cells were isolated from PBMCs of donors using MojoSort human CD8 naïve T cell isolation Kit (BioLegend) according to the standard manufactures. 1 × 10^5^ cells were activated in 96‐well round‐bottom plates using Human CD3/CD28 Stimulation Beads (BioLegend) in RPMI1640 complete medium for 48 h. Then phenotypes of activated cells were evaluated by surface staining and analyzed with the Fortessa instrument and FlowJo software.

### Generation of Bone Marrow Chimera

To generate bone marrow chimeric mice, CD45.1 mice were exposed to 8 Gy of X‐ray radiation. After 24 h, 8 × 10^6^ BMCs, consisting of 4 × 10^6^ wild‐type (CD45.1^+^) and 4 × 10^6^ *Siglecg^−/−^* (CD45.2^+^) BMCs, were injected i.v. into irradiated mice. Eight weeks after reconstitution, mixed bone marrow chimaera mice were inoculated with MC38‐OVA cells or infected with LM‐OVA for further analysis.

### Bacterial Infection and Quantification

Virulent and attenuated recombinant *L. monocytogenes* that secrete OVA protein (LM‐OVA and *∆actA* LM‐OVA) were described previously.^[^ ^77^ ^]^ Recipient mice (CD90.1^+^) were injected of *Siglecg^Gfp/+^* (CD90.2^+^) OT‐I cells and infected with 1 × 10^6^ *∆actA* LM‐OVA or 1 × 10^5^ LM‐OVA 1 day later. The frequency of Siglec‐G^+^ OT‐I cells in PBMC was detected every 2 days. In the sixth day, the Siglec‐G^−^ and Siglec‐G^+^ OT‐I cells were isolated from the spleen to undergo RNA‐seq, and the cytotoxicity of these two subsets was detected. The TNF‐α, IFNγ, and Granzyme B production and proliferation potential were detected in the sixth day. Chimera mice were infected with 1 × 10^6^ *∆actA* LM‐OVA and antigen‐specific CD8^+^ T cells and IFN‐γ production was detected in PBMC, dLN, and spleen in the eighth day. Bacteria in the spleen and liver were measured as previously described.^[^ ^77^ ^]^

### Cytotoxicity Assays

Isolated CD8^+^ T cells from infection models or activated OT‐I CD8^+^ T cells or CAR T cells were plated with MC38‐OVA or MC38‐ hCLDN18.2 cells at different CTL‐to‐target ratios in round‐bottomed 96‐well plates. Cultures were then incubated at 37 °C, 10% CO_2_ for 5 or 8 h (short‐term killing). MC38‐OVA or MC38‐ hCLDN18.2 cells were labeled by CellTrace Violet previously, enabling their distinction from effector cells. The percentage of tumor cell apoptosis was determined using APC Annexin V Apoptosis Detection Kit II (BD Biosciences) following the manufacturer's instructions. Long‐term killing assays were imaged in the IncuCyte S3 Live Cell Analysis System (Sartorius). Isolated CD8^+^ T cells from infection models or activated CD8^+^ T cells were mixed with MC38‐OVA in round‐bottomed 96‐well plates. Plates were scanned using a 4X objective lens in the brightfield and analyzed using IncuCyte S3 v2018b software (Sartorius) to determine the loss of target cells over time based on the decrease of cell intensity.

### Metabolic Assays

A Seahorse XF‐96e extracellular flux analyzer (Seahorse Bioscience, Agilent) was used to determine the metabolic profile of cells. T cells were plated (2 × 10^5^ cells well^−1^) onto cell culture microplates coated with poly‐lysine. Mitochondrial perturbation experiments were carried out using the MitoStress Test Kit by the sequential treatment with oligomycin (Oligo, 1 µm), FCCP (1.5 µm), and R/A (Rotenone A + Antimycin, 0.1 µm). Glycolytic Rate Assay was performed by the sequential injection of Rotenone A + Antimycin A (0.5 µm) and 2‐deoxy*‐D*‐glucose (2‐DG, 50 nm) to discriminate between the glycolysis‐ and mitochondrial‐dependent PER. Oxygen consumption rates (OCR, pmol min^−1^) and proton efflux rate (PER, pmol min^−1^) were monitored in real time after the injection of each compound.

### SHP2 Knockdown in T Cells by shRNA

Shp2 knockdown was performed by lentiviral expression of short hairpin RNA (shRNA) targeting mouse Shp2 (shShp2, 5′‐TTGAGACCAAGTGCAACAATT‐3′; shScramble, 5′‐ CCTAAGGTTAAGTCGCCCTCG‐3′). Lentiviruses were generated in HEK293T cells by co‐transfecting with shRNA pLKO.1‐GFP plasmids and the packaging plasmids pMD2G and psPAX2. Spleen OT‐I cells were isolated from *Siglecg* ^−/−^ (CD45.2^+^) and WT (CD45.1^+^) mice and cultured overnight with T cell activation/expansion kit (Miltenyi Biotec) in the presence of 10 ng ml^−1^ human IL‐2 and 0.2 ng ml^−1^ murine IL‐7. Subsequently, they were spun together with lentivirus onto RetroNectin‐coated (10 µg ml^−1^) 24‐well plates (Takara Bio) and incubated overnight prior to the second viral transduction. Four days later, GFP^+^ cells were sorted, mixed, and transferred into recipient mice (CD90.1^+^) that had been injected with LM‐OVA one day prior.

### Adoptive T Cell and CAR‐T Cell Therapy

For OT‐I CD8^+^ T cells transfer experiments, naïve CD8^+^ T cells obtained from spleens of OT‐I mice and stimulated with 250 ng mL^−1^ OVA peptide for 48 hours in the U‐bottom 96‐well plates. CD45.1 mice (6–8 weeks) were subcutaneously inoculated with 1 × 10^6^ MC38‐OVA cells. Twelve days later, 1 × 10^6^ activated OT‐I CD8^+^ T cells were intravenously transferred into these tumor‐bearing mice for further tumor growth and flow cytometry analysis on day 12 post‐transfer.

For murine CAR T cell transfer experiments, CAR T cells were generated as previously described.^[^ ^78^ ^]^ Briefly, spleen T cells were isolated and cultured overnight with a T cell activation/expansion kit (Miltenyi Biotec) in the presence of 10 ng ml^−1^ human IL‐2 and 0.2 ng ml^−1^ murine IL‐7 in complete RPMI 1640 medium. Lentiviruses encoding a second‐generation CAR targeting hCLDN18.2 purchased from Sanyou Bio spun together with T cells onto RetroNectin‐coated (10 µg ml^−1^) 24‐well plates (Takara Bio) and incubated overnight prior to the second viral transduction. T cells were maintained in IL‐2/IL‐7 containing complete media and used 7–8 days after transduction.

### Immunoblotting

Total proteins from cells were extracted with cell lysis buffer (Cell Signaling Technology) in the presence of a protease inhibitor cocktail (Roche). The protein concentration was measured with a BCA protein assay kit (ThermoFisher Scientific) and equalized with the lysis buffer. An equal amount of the extracts was subject to immunoblot assays using the BIO‐RAD electrophoresis system.

### Real‐Time Quantitative PCR

Total RNA was extracted from isolated or cultured cells with TRIzol reagent (ThermoFisher Scientific) according to the manufacturer's instructions. RNA was reverse transcribed with Oligo (dT) primer for mRNA into cDNA with M‐MLV Reverse Transcriptase (TaKaRa). RNA expression was quantified by real‐time PCR with SYBR RT‐PCR Kit (TaKaRa) and normalized to β‐actin.

### Lactate Dehydrogenase Activity Assay

Lactate dehydrogenase (LDH) activity was detected by the Lactate Dehydrogenase Activity Assay Kit (Sigma). In brief, CD8^+^ T cells were homogenized on ice in 500 µL of cold LDH Assay buffer. The soluble fraction was used to test LDH activity.

### Statistical Analysis

Statistical analyses were calculated using GraphPad Prism as indicated in the figure legends. For comparing tumor growth curves, the two‐way analysis of variance (ANOVA) with Sidak's multiple comparisons test was used. For survival analysis, a Log‐rank (Mantel–Cox) test was used. Data are presented as mean ± SD. The value of *p* < 0.05 was considered statistically significant. *p < 0.05, **p < 0.01, ***p < 0.001, and ****p < 0.0001.

## Conflict of Interest

The authors declare no conflict of interest.

## Author Contributions

S.Y., C.L., and X.S. contributed equally to this work. S.Y., C.L., and X.S. conceived the study, designed the experiments, and interpreted the results. S.Y. and C.L. performed most of the experiments. X.S. performed bioinformatics analyses on public scRNA‐seq and TCGA databases. Y.H., S.Y., L.C., and Y.W. assisted with the establishment of tumor mouse models. J.C. and S.L. assisted in the immunoblot experiments. G.Q. and M.S. contributed to flow cytometry experiments. C.Q. provided technical assistance. S.Y., C.L., and X.S. wrote the paper. S.X., Y.Y., and Z.Z. supervised biological research and the study design.

## Supporting information



Supporting Information

## Data Availability

The data that support the findings of this study are available from the corresponding author upon reasonable request.
