# Supplementary material for: Siglec‐G Suppresses CD8+ T Cells Responses through Metabolic Rewiring and Can be Targeted to Enhance Tumor Immunotherapy
Source: Adv Sci (Weinh). 2024 Oct 7;11(45):2403438. doi: 10.1002/advs.202403438 (PMC11615767; doi:10.1002/advs.202403438)
Supplement: Supplementary file 1 — Supporting Information [file ADVS-11-2403438-s001.pdf]

ADVANCED SCIENCE
Open Access

Supporting Information

for *Adv. Sci.*, DOI 10.1002/advs.202403438

Siglec-G Suppresses $CD8^+$ T Cells Responses through Metabolic Rewiring and Can be Targeted to Enhance Tumor Immunotherapy

Shenhui Yin, Chunzhen Li, Xin Shen, Guanyu Yu, Likun Cui, Yunyang Wu, Yixian He, Shu Yu, Jie Chen, Shaoteng Lu, Guifang Qiu, Mengqi Song, Cheng Qian, Zui Zou, Yizhi Yu* and Sheng Xu**

Supporting Information

Siglec-G Suppresses CD8$^+$ T Cells Responses through Metabolic Rewiring and Can be Targeted to Enhance Tumor Immunotherapy

Shenhui Yin[1,5], Chunzhen Li[1,5], Xin Shen[1,5], Guanyu Yu[2], Likun Cui[1], Yunyang Wu[1], Yixian He[1], Shu Yu[1], Jie Chen[1], Shaoteng Lu[1], Guifang Qiu[1], Mengqi Song[1], Cheng Qian[1], Zui Zou[3,4], Yizhi Yu[1]* and Sheng Xu[1]**

[1]National Key Laboratory of Immunity & Inflammation, Naval Medical University/Second Military Medical University, Shanghai 200433, China

[2]Department of Colorectal Surgery, Changhai Hospital, Naval Medical University, Shanghai 200433, China.

[3]School of Anesthesiology, Naval Medical University, Shanghai 200433, China

[4]Faculty of Anesthesiology, Changhai Hospital, Naval Medical University, Shanghai 200433, China

[5]These authors contributed equally

*Correspondence:zouzui@smmu.edu.cn;yuyz@immunol.org; xusheng@immunol.org

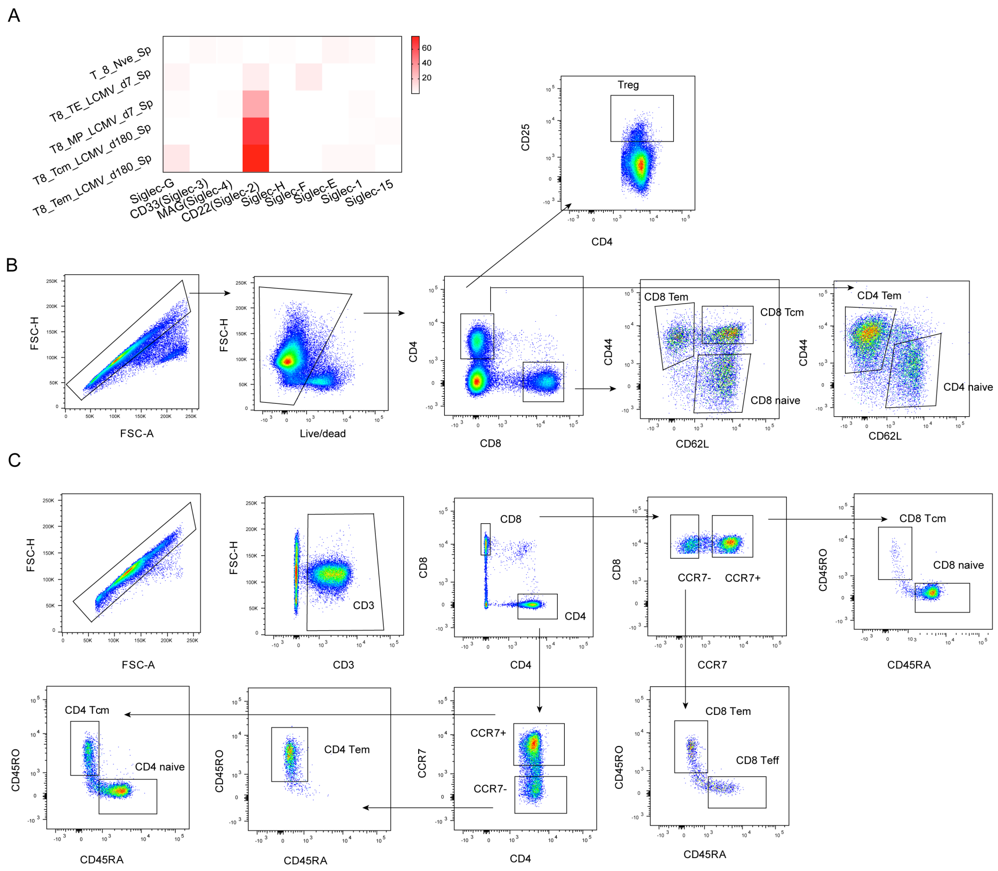

Supplementary Figure S1. Further Characteristic Siglec-G expression and gating strategy for different types of immune cells; related to Figure 1.

A) Heatmap showing Siglecs family genes expression in LCMV infection model from public database Immunological Genome Project (ImmGen). B) Gating strategy for $CD8^+$ effector memory T cells ($CD8^+$ $CD44^{hi}$ $CD62L^{lo}$), $CD8^+$ central memory T cells ($CD8^+$ $CD44^{hi}$ $CD62L^{hi}$), $CD8^+$ naive T cells ($CD8^+$ $CD44^{lo}$ $CD62L^{hi}$), $CD4^+$ effector memory T cells ($CD4^+$ $CD44^{hi}$ $CD62L^{lo}$), $CD4^+$ naive T cells ($CD4^+$ $CD44^{lo}$ $CD62L^{hi}$) and Treg cells ($CD4^+$ $CD25^{hi}$) in murine spleen. C) Gating strategy for $CD8^+$ effector T cells ($CD8^+$ $CCR7^{lo}$ $CD45RA^{hi}$ $CD45RO^{lo}$), $CD8^+$ effector memory T cells ($CD8^+$ $CCR7^{lo}$ $CD45RA^{lo}$ $CD45RO^{hi}$), $CD8^+$ central memory T cells ($CD8^+$ $CCR7^{hi}$ $CD45RA^{lo}$ $CD45RO^{hi}$), $CD8^+$ naive T cells ($CD8^+$ $CCR7^{hi}$ $CD45RA^{hi}$ $CD45RO^{lo}$), $CD4^+$ effector memory T cells ($CD4^+$ $CCR7^{lo}$ $CD45RA^{lo}$ $CD45RO^{hi}$), $CD4^+$ central memory T cells ($CD4^+$ $CCR7^{hi}$ $CD45RA^{lo}$ $CD45RO^{hi}$) and $CD4^+$ naïve T cells ($CD4^+$ $CCR7^{hi}$ $CD45RA^{hi}$ $CD45RO^{lo}$) in human PBMC.

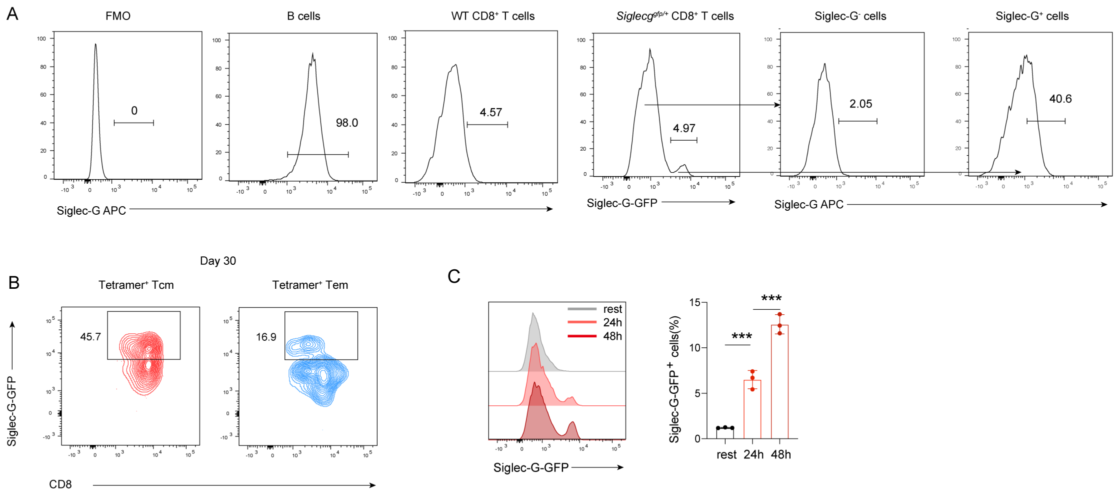

Supplementary Figure S2. Expression of Siglec-G in Tcm and Tem in vivo; related to Figure 1.

A) Splenocytes were isolated from WT or *Siglecg*$^{Gfp/+}$ animals and analyzed for Siglec-G expression utilizing a monoclonal antibody to Siglec-G (clone SH2.1) and the expression of GFP in *Siglecg*$^{Gfp/+}$ animal. B) *Siglecg*$^{Gfp/+}$ animals were infected with LM-OVA and the expression of Siglec-G in antigen-specific $CD8^+$ Tcm and Tem were analyzed 30 days later. C) Murine naïve $CD4^+$ T cells were activated with BMDCs supplemented with 100 µg mL^{-1} OVA and Siglec-G expression were detected over time (n = 3). Representative data are shown from three independent experiments. The error bar represents mean ± SD. Statistical significance was determined by one-way ANOVA with multiple comparisons (C). ***$p < 0.001$.

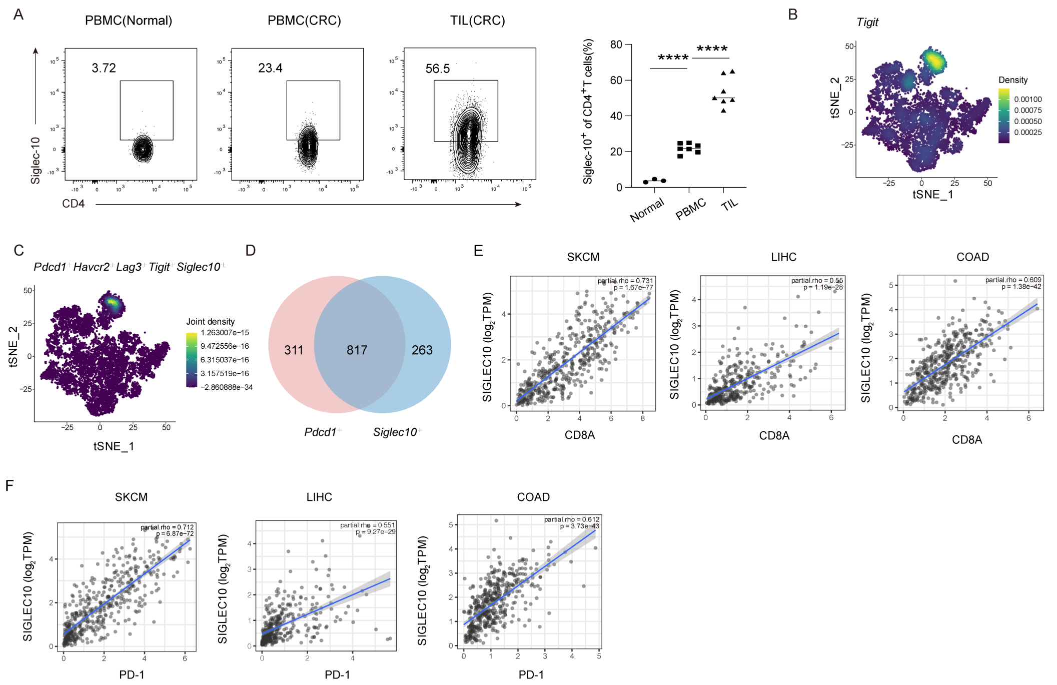

Supplementary Figure S3. Further characterization of Siglec-10 in tumors; related to Figure 2.

A) Siglec-10 expression in peripheral and tumor-infiltrating $CD4^+$ T cells (n =3--7). B, C) t-Distributed Stochastic Neighbor Embedding (t-SNE) plots showing the rank-normalized expression of the $CD8^+$ T cell dysfunction signature and *Tigit* in tumor-infiltrating single $CD8^+$ T cells (GSE231559). D) Venn diagrams showing the number of PD-1 expressing tumor-infiltrating single $CD8^+$ T cells compared to Siglec-10 expressing cells in GSE231559. E, F) TIMER analysis of the relationship between Siglec-10 and CD8 or PD-1 in human cancer. Representative data are shown from three independent experiments. The error bar represents mean ± SD. Statistical significance was determined by one-way ANOVA with multiple comparisons (A). ****$p < 0.0001$.

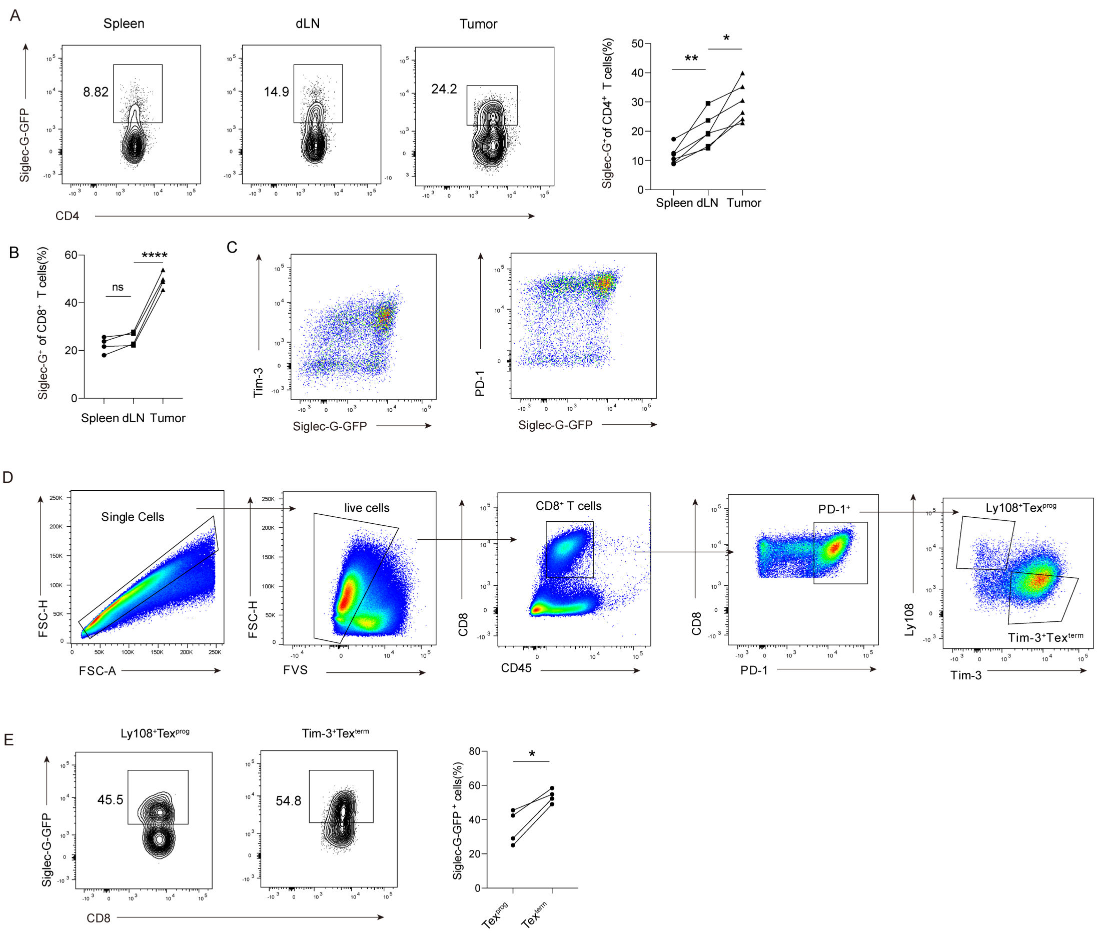

Supplementary Figure S4. Further characterization of murine Siglec-G expression in tumors; related to Figure 2.

A) Siglec-G expressing splenocytes, dLNs or tumor-infiltrating $CD4^+$ T cells ($n = 6$). B) *Siglecg*$^{Gfp/+}$ reporter mice were injected s.c. with B16 cells and analyzed as described as Figure 2. The frequency of Siglec-G^+ $CD8^+$ T cells and $CD4^+$ T cells in spleen, dLNs and tumor ($n = 4$). C) Siglec-G and PD-1 or Tim-3 expression in tumor-infiltrating $CD8^+$ T cells. D) Gating strategy for $Ly108^+Tex^{prog}$ ($CD45^+CD8^+PD\text{-}1^+Ly108^+Tim\text{-}3^-$) and $Tim\text{-}3^+Tex^{term}$ ($CD45^+CD8^+PD\text{-}1^+Ly108^-Tim\text{-}3^+$) in *Siglecg*$^{Gfp/+}$ reporter mice inoculated with MC38 cells. E) The expression of Siglec-G in $Ly108^+Tex^{prog}$ and $Tim\text{-}3^+Tex^{term}$ in *Siglecg*$^{Gfp/+}$ reporter mice inoculated with MC38 cells ($n = 4$). Representative data are shown from three independent experiments. The error bar represents mean ± SD. Statistical significance was determined by one-way ANOVA with multiple comparisons (A, B), paired Student's *t*-test (E). $*p < 0.05$, $**p < 0.01$, and $****p < 0.0001$.

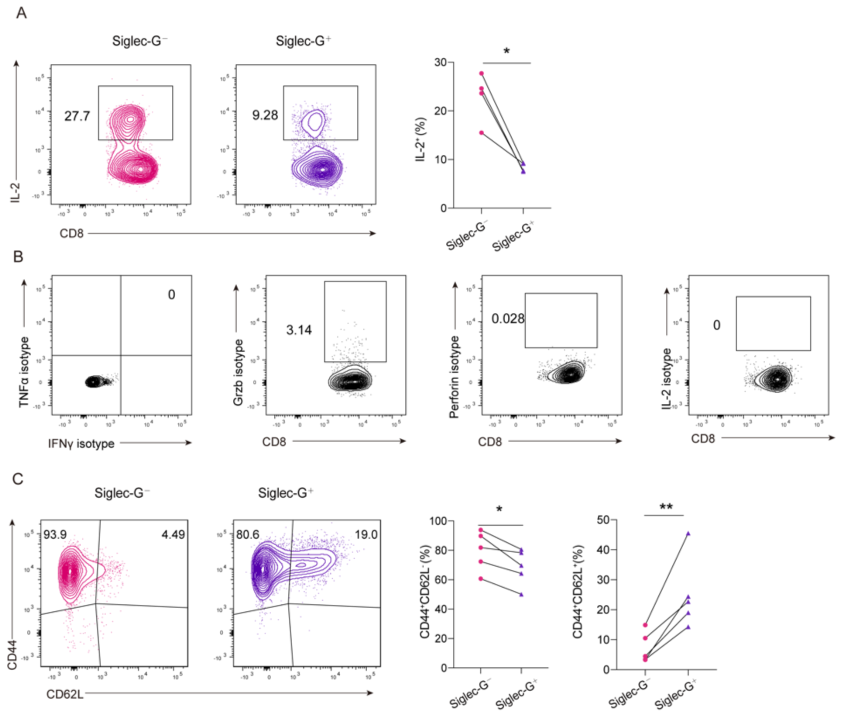

Supplementary Figure S5. Further characterization of murine Siglec-G function in tumors; related to Figure 2.

A) Intracellular IL-2 production in the Siglec-G$^-$ and Siglec-G$^+$ tumor-infiltrating CD8$^+$ cells (n = 4). B) Isotype control for intracellular staining. C) CD44 and CD62L expression in the Siglec-G$^-$ and Siglec-G$^+$ tumor-infiltrating CD8$^+$ cells (n = 5). Representative data are shown from three independent experiments. The error bar represents mean ± SD. Statistical significance was determined by paired Student's t test (A and C). $^*p < 0.05$, $^{**}p < 0.01$.

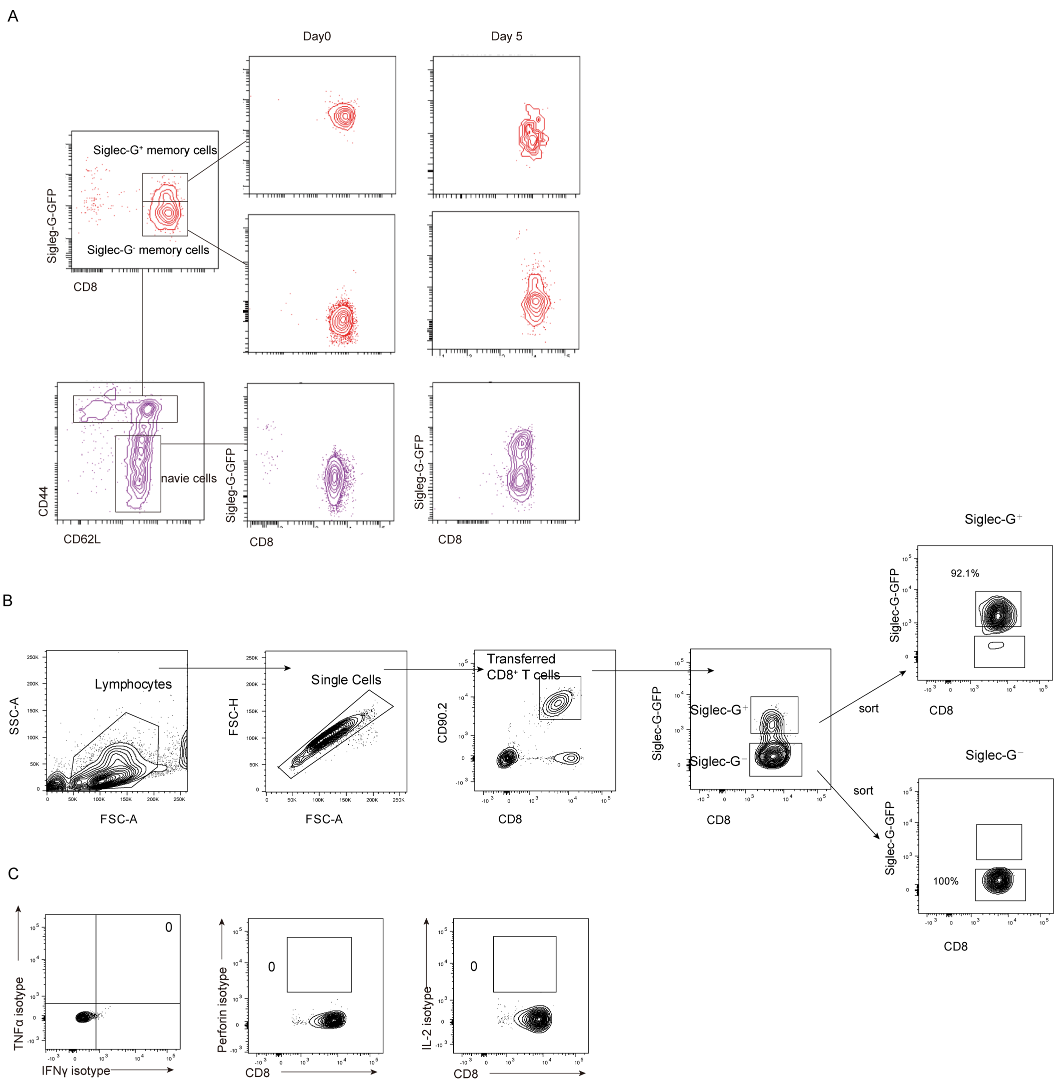

Supplementary Figure S6. The expression change of Siglec-G in different CD8$^+$ T cells after infection and gating strategy of transferred cells; related to Figure 3.

A) The expression change of Siglec-G after infection in vivo in naïve CD8$^+$ T cells, Siglec-G$^-$ memory cells and Siglec-G$^+$ memory cells. B) Gating strategy for isolation of transferred Siglec-G$^-$ OT-I subset (CD90.2$^+$ CD8$^+$ Siglec-G-GFP$^-$) and Siglec-G$^+$ OT-I subset (CD90.2$^+$ CD8$^+$ Siglec-G-GFP$^+$) from spleen, and the purity of these two subsets were reconfirmed. C) Isotype control for intracellular staining. Representative data are shown from three independent experiments.

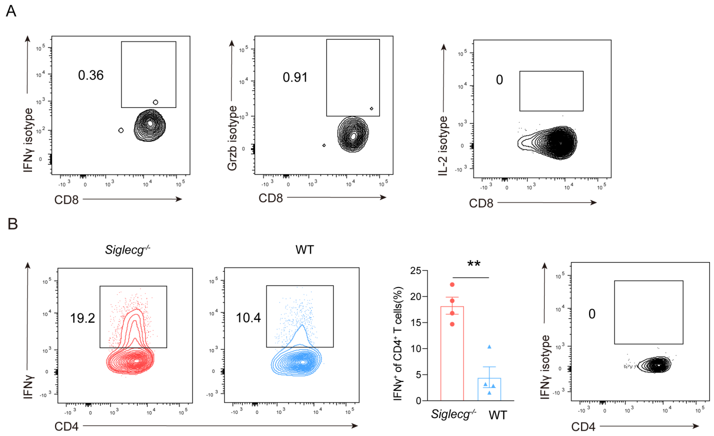

Supplementary Figure S7. Siglec-G deficiency promotes the differentiation of CD4$^+$ T cells into Th1 cells; related to Figure 4.

A) Isotype control for intracellular staining. B) CD4$^+$ T cells from *Siglecg*$^{-/-}$ and WT littermate mice were activated with BMDCs and anti-CD3 mAb supplement with anti-mouse IL4 (10 μg mL^{-1}) and r-m IL12 (20 ng mL^{-1}). The production of IFN-γ was detected 3 days after differentiation. Representative data are shown from three independent experiments. The error bar represents mean ± SD. Statistical significance was determined by unpaired Student's *t* test (B). $^{**}p < 0.01$.

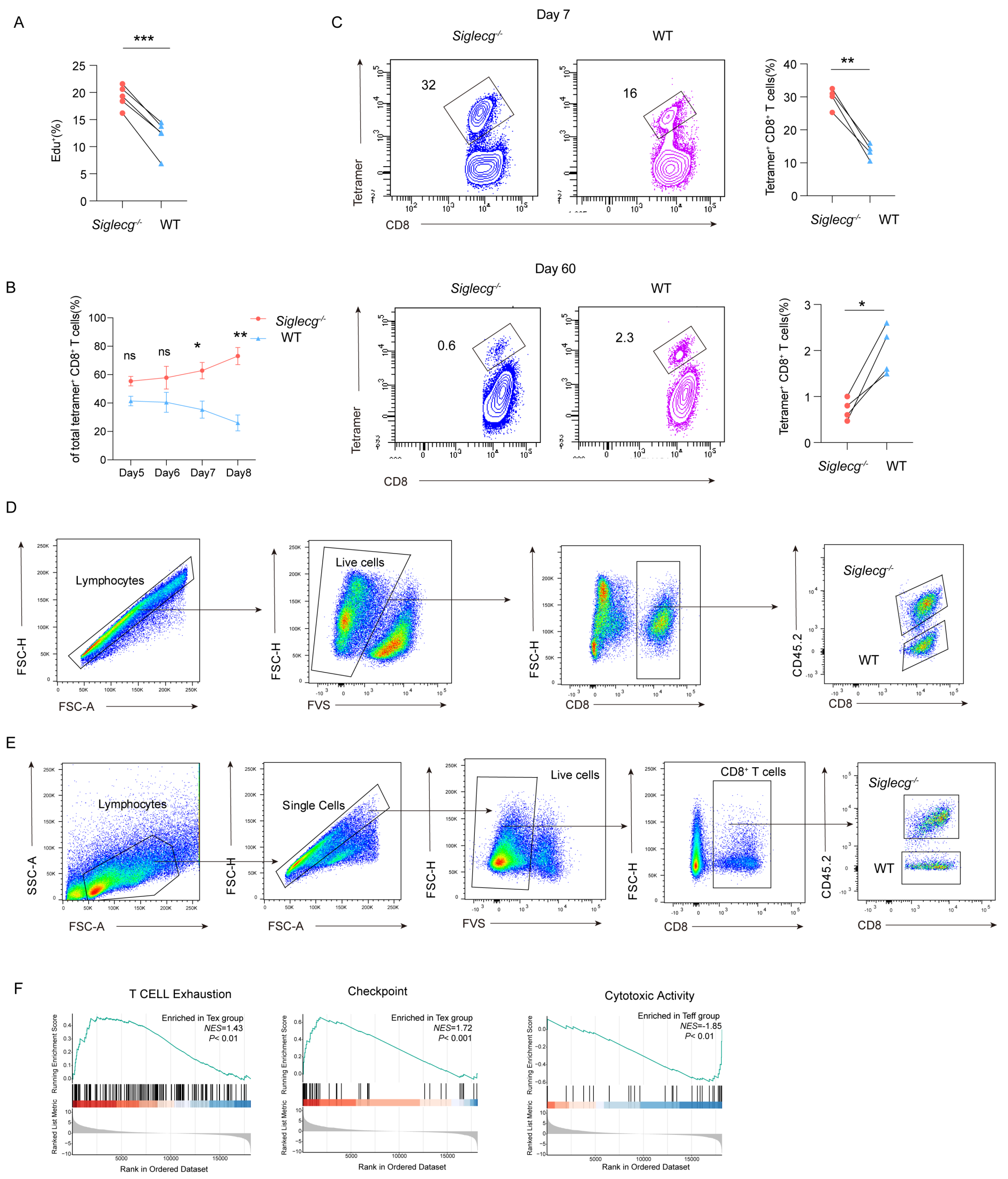

Supplementary Figure S8. Siglec-G deficiency promotes enhanced T cells effector function in tumor and infection but decreased memory potential; related to Figure 5.

A) The fast proliferative proportion of *Siglecg*$^{-/-}$ and WT CD8^{+} T cells from LM-OVA infected chimera mice (n = 5). B) The proportion of *Siglecg*$^{-/-}$ and WT antigen-specific T cells among total antigen-specific T cells (n = 3). C) Antigen -specific CD8^{+} T cells in spleens in chimeric mice infected with LM-OVA after 7 days and 60 days. D, E) Gating strategy for *Siglecg*$^{-/-}$ and WT CD8^{+} T cells in chimeric mice bearing tumor or infected with LM-OVA. F) GSEA analysis on gene pathways associated with T cell exhaustion, checkpoint and cytotoxic activity pathways in *Siglecg*$^{-/-}$ Tex cells isolated

from tumor bearing mice compared to *Siglecg*$^{-/-}$ Teff cells isolated from LM-OVA infected mice. Representative data are shown from three independent experiments. The error bar represents mean ± SD. Statistical significance was determined by paired Student's *t* test (A-C). *$p < 0.05$, **$p < 0.01$ and ***$p < 0.001$.

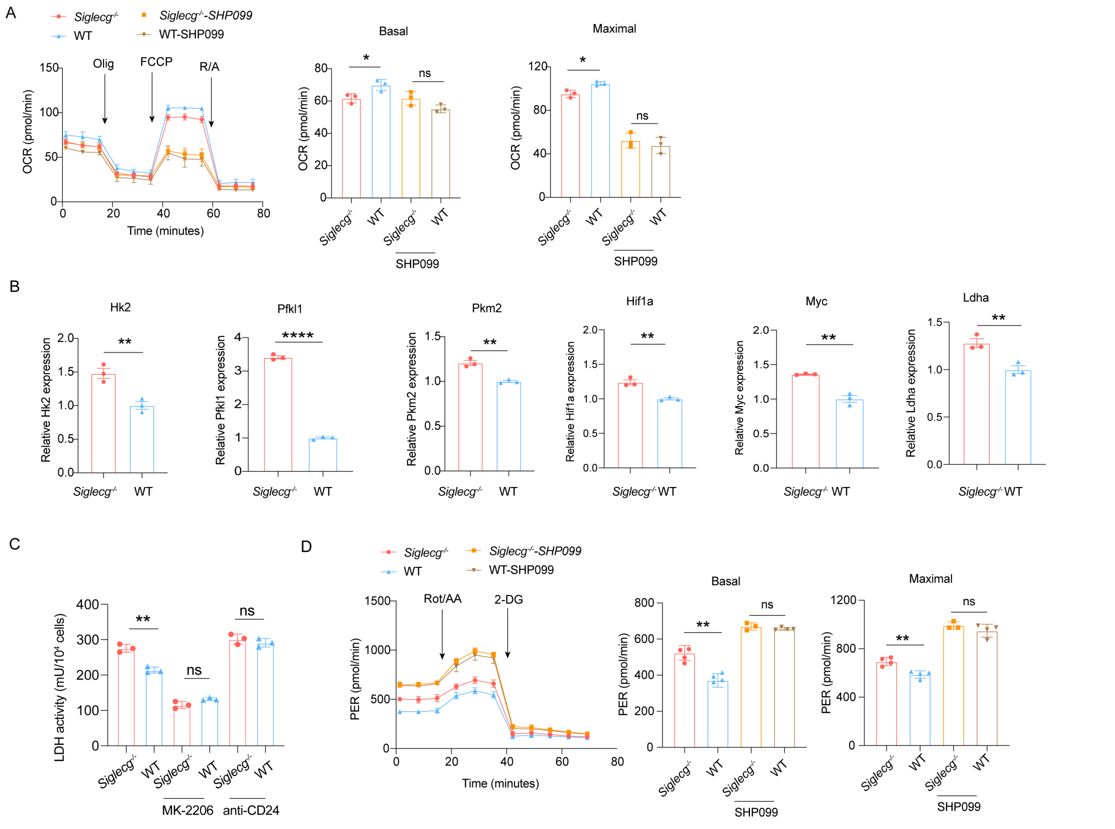

Supplementary Figure S9. Metabolic programs of *Siglecg*$^{-/-}$ and WT CD8^{+} T cells; related to Figure 6.

A) Seahorse analysis of mitochondrial oxygen consumption rate (OCR) of activated *Siglecg*$^{-/-}$ and WT CD8^{+} T cells or treated with inhibitor of 10 μM SHP2 (SHP099) for 2 h. B) Q-PCR analysis of the expression of indicated genes. C) Lactate dehydrogenase (LDH) activity in *Siglecg*$^{-/-}$ and WT CD8^{+} T cells, or treated with 10 μM AKT inhibitor MK-2206 or anti-CD24 neutralizing antibody. D) Seahorse analysis of glycolytic proton efflux rate (glycoPER) of activated *Siglecg*$^{-/-}$ and WT CD8^{+} T cells or treated with SHP099 for 2 h. Representative data are shown from three independent experiments. The error bar represents mean ± SD. Statistical significance was determined by one-way ANOVA with multiple comparisons (A, C and D) and unpaired Student's *t* test (B). *$p < 0.05$, **$p < 0.01$ and ****$p < 0.0001$.

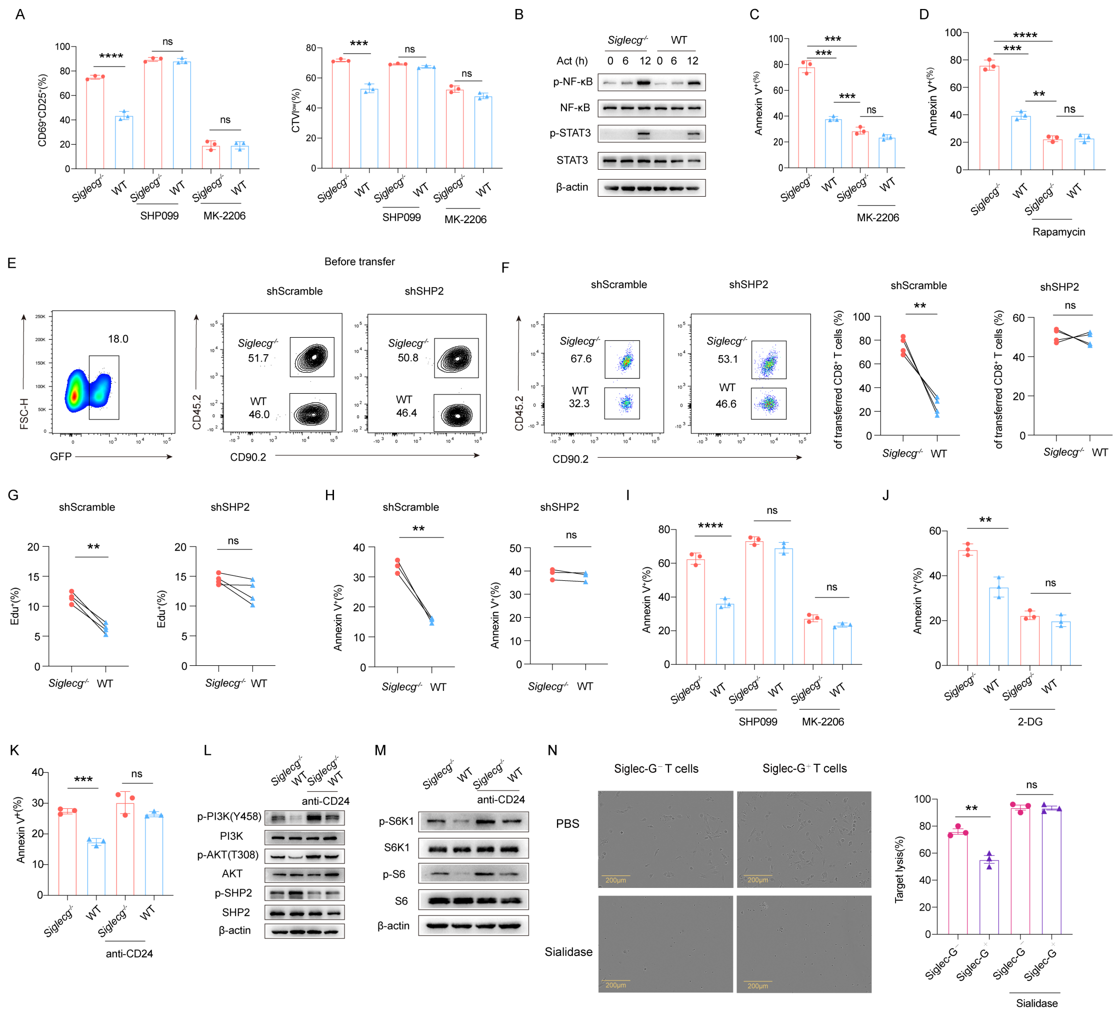

Supplementary Figure S10. SHP2-PI3K-AKT pathway in Siglec-G mediated CD8⁺ T cells function regulation; related to Figure 6.

A) Activated *Siglecg*$^{-/-}$ and WT CD8⁺ T cells were treated with 10 μM SHP099 or 10 μM MK-2206 or not. CD25 and CD69 levels indicated activation and CTV^{low} proportion indicated proliferation were determined by flow cytometry. B) Immunoblotting showing phosphorylated and total NF-κB and STAT3 in activated *Siglecg*$^{-/-}$ and WT CD8⁺ T cells. C) The percentage of Annexin V⁺ MC38-OVA cells co-cultured with activated *Siglecg*$^{-/-}$ or WT OT-I CD8⁺ T cells, treated with glycolysis inhibitor 10 μM MK-2206 or not for 2 h. D) The percentage of Annexin V⁺ MC38-OVA cells co-cultured with activated *Siglecg*$^{-/-}$ or WT OT-I CD8⁺ T cells, treated with 1 μM mTOR inhibitor Rapamycin or not for 4 h. E) Activated CD45.2⁺ *Siglecg*$^{-/-}$ and CD45.1⁺ WT OT-I cells were sorted by GFP after transduction with shScramble-GFP or shSHP2-

GFP lentivirus. An equal proportion of *Siglecg*$^{-/-}$ (CD45.2$^+$) and WT (CD45.1$^+$) CD8$^+$ T cells, or *Siglecg*$^{-/-}$ and WT CD8$^+$ T cells with SHP2 knockdown were transferrd into recipient mice (CD90.1$^+$) injected with LM-OVA. F) The proportion of *Siglecg*$^{-/-}$ and WT CD8$^+$ T cells in the spleen 6 days later (n = 4). G) The proliferative proportion of these cell types (n = 4). H) The apoptosis of MC38-OVA cells co-cultured with *Siglecg*$^{-/-}$ and WT OT-I cells isolated above (n = 3). I) *Siglecg*$^{-/-}$ and WT effector OT-I cells were isolated from LM-OVA infected mice, treated with 10 μ_M SHP099 or MK-2206, and co-cultured with MC38-OVA cells. The cytotoxicity was assessed by the percentage of Annexin V$^+$ MC38-OVA cells. J) The apoptosis of MC38-OVA cells co-cultured with *Siglecg*$^{-/-}$ and WT OT-I cells treated with 2 m_M 2-DG or not. K) The percentage of Annexin V$^+$ MC38-OVA cells co-cultured with *Siglecg*$^{-/-}$ and WT OT-I CD8$^+$ T cells, treated with anti-CD24 neutralizing antibodies or not. L, M) Immunoblotting analysis of the indicated proteins in *Siglecg*$^{-/-}$ and WT CD8$^+$ T cells treated with anti-CD24 neutralizing antibodies or not. N) Short-term killing assay showing MC38-OVA cell lysis after co-cultured with sorted Siglec-G$^-$ and Siglec-G$^+$ OT-I subsets , MC38-OVA treated with sialidase or not. Representative data are shown from three independent experiments. The error bar represents mean ± SD. Statistical significance was determined by one-way ANOVA with multiple comparisons (A, C, D, I, J, K and N) and paired Student's t test (F-H). $^{**}p < 0.01$, $^{***}p < 0.001$, and $^{****}p < 0.0001$.

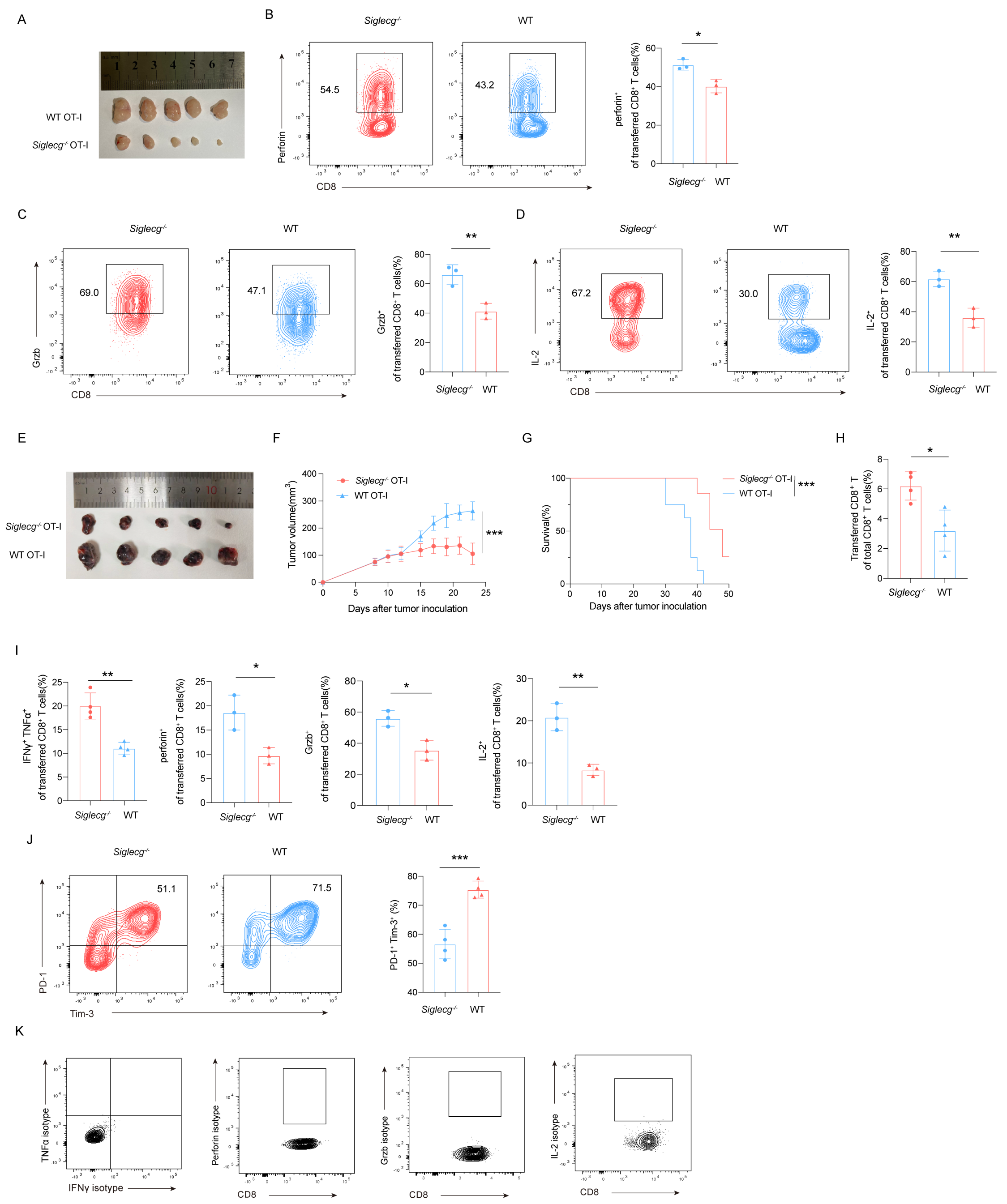

Supplementary Figure S11. Siglec-G deletion in CD8$^+$ T cells enhances anti-tumor immunity; related to Figure 7.

A) Tumor of MC38-OVA-bearing mice treated with activated *Siglecg*$^{-/-}$ and WT CD8$^+$ T cells. B-D) Intracellular Perforin (B), Granzyme B (C) and IL-2 (D) production of transferred *Siglecg*$^{-/-}$ and WT CD8$^+$ T cells from MC38-OVA-bearing mice. E-G) Tumor size (E), tumor growth (F) and survival (G) of B16-OVA-bearing mice. H) The proportions of adoptively transferred *Siglecg*$^{-/-}$ and WT OT-I CD8$^+$ T cells in total intratumor CD8$^+$ T cells from B16-OVA-bearing mice (n = 5). I) Intracellular IFN-γ

and TNF-α expression, Perforin, Granzyme B and IL-2 production on adoptively transferred *Siglecg*$^{-/-}$ and WT OT-I CD8^{+} T cells from B16-OVA-bearing mice. J) PD-1 and Tim-3 expression on transferred *Siglecg*$^{-/-}$ and WT OT-I CD8^{+} T cells. K) Isotype control for intracellular staining. Representative data are shown from three independent experiments. The error bar represents mean ± SD. Statistical significance was determined by unpaired Student's *t* test (B-D, H-J), two-way ANOVA with multiple comparisons (F) and a Log-rank (Mantel-Cox) test (G). *$p < 0.05$, **$p < 0.01$ and ***$p < 0.001$.

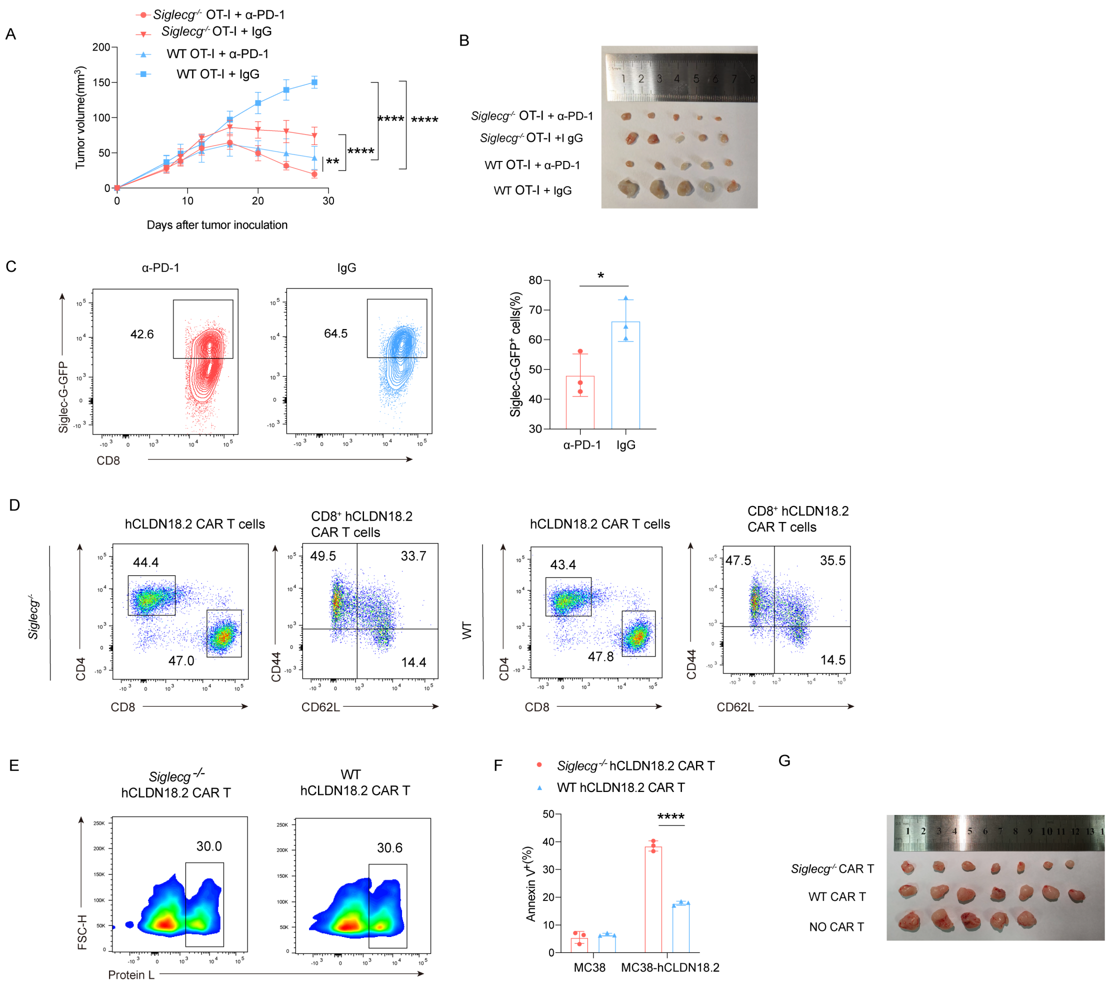

Supplementary Figure S12. Siglec-G deficiency has synergistic effect with the anti-PD-1 antibody and enhances CAR T cell cytotoxicity in vitro; related to Figure 7. A and B) Tumor bearing mice were treated with activated *Siglecg*$^{-/-}$ and WT OT-I cells at 12th day, plus with α-PD-1 or α-IgG (200 μg) every three days. Tumor growth were measured every 2-3 days and tumor were dissected at last day (*n* = 5) C) The expression of Siglec-G under the treatment of α-PD-1 or α-IgG. D) CAR T cells generated from *Siglecg*$^{-/-}$ and WT littermate mice splenocytes were assessed for the proportions of CD4^{+} and CD8^{+} total T cells as well as CD44^{hi}CD62L^{hi} central memory and CD44^{hi}CD62L^{lo} effector CD8^{+} T cells. E) CAR transduction efficiency was detected by Protein L. F) CAR T cells were cultured with MC38 or MC38-hCLDN18.2 cells for 8 hours and quantification of the percentage of Annexin V^{+} tumor cells (relative to total cells). G) Tumor of MC38- hCLDN18.2-bearing mice treated with activated *Siglecg*$^{-/-}$

and WT CAR T cells. Representative data are shown from three independent experiments. The error bar represents mean ± SD. Statistical significance was determined by two-way ANOVA with multiple comparisons (A) and unpaired Student's *t* test (C and F). $*p < 0.05$, $**p < 0.01$, and $****p < 0.0001$.

Supplementary Table S1:

Gene sets involved in GSEA and GSVA analysis.

Name	Accession/DOI	Journal (year)	[illegible]
GOBP_OXIDATIVE_PHOSPHORYLATION	http://www.gsea-msigdb.org/gsea/msigdb/mouse/geneset/GOBP_OXIDATIVE_PHOSPHORYLATION	-	[illegible]
T CELL Exhaustion	https://doi.org/10.1038/s41467-020-20019-0	Nature Communications (2020)	[illegible]
Cytotoxic activity	https://doi.org/10.1038/s41467-020-20019-0	Nature Communications (2020)	[illegible]
Checkpoint	https://doi.org/10.1016/j.cell.2014.12.033	Cell (2014)	[illegible]
Cytotoxic signature	10.1038/s41467-020-20019-0	Nature Communications (2020)	[illegible]
Activation:Effector function	10.1038/s41591-023-02371-y	Nature Medicine (2023)	[illegible]
IFN-Gamma	10.1172/JCI91190	The Journal of Clinical Investigation (2017)	[illegible]
